# TSG6 promotes epithelial-mesenchymal transition and tumor-associated macrophage polarization through Smad2/3 and MAPK signaling by facilitating TSG6-CD44-TGFβR1 or EGFR complex formation

**DOI:** 10.7150/ijbs.115097

**Published:** 2025-07-24

**Authors:** Hyun-Ji Oh, Ga-Hong Min, Da-Eun Kim, Sol-bi Shin, Hyungshin Yim

**Affiliations:** Department of Pharmacy, College of Pharmacy, Institute of Pharmaceutical Science and Technology, Hanyang University, Ansan, Gyeonggi-do 15588, Republic of Korea.

**Keywords:** TSG6, invasiveness, tumor-associated macrophages, CD44

## Abstract

TSG6 is highly expressed during PLK1-induced epithelial-mesenchymal transition (EMT). However, the role of TSG6 in the tumor microenvironment (TME) remains poorly understood. We investigate the function and regulatory mechanisms of TSG6 in immune plasticity within the TME of lung adenocarcinoma (LUAD). The simultaneous high expression of TSG6 and PLK1 in LUAD patients was associated with lower survival rates. TSG6 and CD44 were markedly upregulated during EMT driven by TGF-b or active PLK1 in A549 and HCC827 cells. TSG6 treatment enhanced EMT by increasing N-cadherin and phosphorylated Smad2 levels. TSG6 depletion blocked the effects, which was restored upon TSG6 retreatment. Additionally, TSG6 treatment induced polarization of THP-1 monocytes into M2d tumor-associated macrophages (TAMs). In cocultures of THP-1 monocytes with A549 cells expressing TSG6, M2d-inducing factors in A549 cells and M2d markers in THP-1 cells were upregulated. Immunoprecipitation showed that TSG6 binds CD44, enhancing CD44's interaction with TGFbR or EGFR. In TSG6-treated LUAD cells, both total CD44 and its cleaved intracellular domain increased by activating TGFβR1-Smad2/3 and MAPK-ERK1/2-AP-1 pathways. Thus, TSG6 promotes EMT and M2d-TAMs polarization by activating TGFβR1/Smad and MAPK/ERK pathway through direct interaction between CD44 and TGFβR1 or EGFR.

## Introduction

The tumor microenvironment (TME) is a complex environment consisting of cancer cells, blood vessels, extracellular matrix, and immune cells including T cells, B cells, monocytes and dendritic cells 1-3. The interactions between cancer cells and immune cells or blood vessel cells have been studied to understand the cell plasticity in TME 1-3. The composition of TME depends on the tumor type but typically includes immune cells, blood vessels, and extracellular matrix (ECM) 1. During the early stages of tumor growth, mutual relationships between cancer cells and TME components support the survival of cancer cells and development of malignancy 1, 4. Especially, in tumor malignancy, monocytes are closely involved by differentiating into tumor-associated macrophages (TAMs) 5. In the TME, M2-like TAMs reside and functions as anti-inflammatory and tumor-supporting macrophages 5. M2 macrophages are classified into M2a, M2b, M2c, and M2d macrophages, which are stimulated by IL-4/IL-13, lipopolysaccharide/IL-1 receptor, IL-10, and VEGF, respectively 2. Polarized M2 phenotype plays a crucial role in tumor proliferation, metastasis, and immune evasion by expressing anti-inflammatory factors such as IL-10 and transforming growth factor (TGF)-β 2. Mononuclear cells are attracted by cytokines including CCL-2, CSF1, and VEGF and they are typically polarized into TAMs. The polarized M2-like macrophage markers and cytokines such as mannose receptors (CD206), scavenger receptors (CD163), VEGF, and IL-10, exhibit tumor-supporting effects 2.

Tumor necrosis factor (TNF)-stimulated gene-6 (TSG6) is the hyaluronan (HA)-binding protein through its LINK domains 6, 7. HA synthesized by HAS releases into the ECM, which undergoes macromolecular reconstruction through its interacts with HA-binding proteins, including CD44 8. The interaction between HA and CD44 is an instrumental event in regulating inflammation and cancer 9. TSG6 also forms an oligomer with HA to regulate the cross-linking of the HA and modify the ECM 10. Moreover, both full-length TSG6 and its LINK domain enhance the binding of HA to CD44 on the cell surface, which results in leukocyte rolling and their recruitment to sites of inflammation 11. The precise role of TSG6 in TME has remained vague, because it is upregulated in the pro-inflammatory environment 6. Interestingly, it is also upregulated in several cancer patients including lung cancer 12, colorectal cancer (CRC) 13, head and neck cancer 14, and urothelial carcinomas 15 with poor prognosis. Notably, its overexpression significantly increased in metastatic CRC patients, activating JAK2-STAT3 signals for metastasis 13. Furthermore, we recently found that *TNFAIP6* (encoding TSG6) is the most highly expressed gene in active PLK1-driven EMT of non-small cell lung cancer (NSCLC) cells 12.

PLK1, a mitotic serine/threonine kinase, serves as a master regulator of mitosis, controlling processes from centrosome maturation at mitotic entry to mitotic exit through phosphorylation activity 16. Because of its pivotal involvement in cell division, PLK1 contributes to cell proliferation, and its overexpression is positively associated with various cancers and with advanced stages of several malignancies 16-19 including those of the breast 17, prostate 18, and stomach 19. In the context of lung adenocarcinoma, PLK1 promotes the EMT by phosphorylating key factors such as β-catenin 20, vimentin 21, and FoxM1 4. Notably, our previous work demonstrated that the active form of PLK1, phosphorylated at Thr210, drives EMT in NSCLC 12. During this process, NSCLC cells exhibited elevated expression of *TNFAIP6*. Depleting *TNFAIP6* showed its pivotal role in PLK1-mediated EMT, especially in cell migration and invasion 12. Then, we observed that its expression was transcriptionally regulated by phosphorylated β-catenin by PLK1 in NSCLC cells 20. However, the functions of TSG6 in the TME are not clearly understood. Here, we have found that TSG6 facilitates M2d-like macrophage polarization and EMT in the TME of LUAD by activating TGFβR1/Smad and MAPK/ERK signaling through its direct interaction with its receptor CD44, which promotes the complex formation with TGFβR1 or EGFR.

## Materials and methods

### Bioinformatics analysis

The patient data were extracted from an online database (www.kmplot.com) following previous reports 4, 21, 22. In the database, all cancer patients were identified from the Cancer Genome Atlas (http://cancergenome.nih.gov) or the Gene Expression Omnibus (http://www.ncbi.nlm.nih.gov/geo/). To establish the clinical relevance of PLK1 and TNFAIP6 expression to the survival of patients, the database was established using these genes expression data and survival information from 1,152 patients after excluding biased arrays. The samples were split into low and high groups using the median expression of each factor. Hazard ratios (HRs) with 95% confidence intervals. Log-rank *P* were calculated according to the online formulas provided by each database. A log-rank *P* value of < 0.05 was considered statistically significant. In survival analysis, HR represents the ratio of the hazard rates between two levels of an explanatory variable as shown in Table S1.

### Cell culture and treatment

Human monocyte THP-1, lung fibroblast MRC5, lung adenocarcinoma (LUAD) A549, and HCC827 cells were purchased from the KCLB (Seoul, Korea). HEK293T cells were purchased from ATCC (Manassas, VA, USA). A549, HCC827, and THP-1 cells were cultured in RPMI-1640 (Corning Cellgro; Manassas, VA, USA), MRC5 cells were cultured in MEM (Corning Cellgro), and HEK293T cells were cultured in DMEM (Corning Cellgro), supplemented with 10% FBS in the presence of penicillin and streptomycin (Corning Cellgro) in a humidified 5% CO_2_ incubator at 37°C. For TGF-β treatment, cells were treated with 2.5 ng/ml TGF-β for 48 hours based on a previous study 12. Cells were seeded at 2.5×10^4^ cells/ml for 16 hours. For recombinant human TSG6 (rhTSG6) treatment, THP-1 cells were seeded 1.5×10^5^ cells/ml and treated with 200 ng/ml with rhTSG6 for two hours or the indicated duration after 24 hours based on previous studies 23, 24. 10 μM trametinib was applied for 48 hours based on the GI_50_ value in A549 cells 25. All other chemical reagents were purchased from Sigma-Aldrich (St. Louis, MO, USA). For the coculture system, human monocytes THP-1 cells and A549 cells expressing TSG6 (TSG6-WT) were cultured in RPMI-1640 (Corning Cellgro) supplemented with 10% FBS in the presence of penicillin and streptomycin (Corning Cellgro) in a humidified 5% CO_2_ incubator at 37 °C.

### Immunoblotting

For cell lysis, lysis buffer (1 mM dithiothreitol, 1 mM EGTA, 50 mM β-glycerophosphate, 0.5% Triton X-100, 20 mM Tris, pH 7.5, 2 mM MgCl_2_, 25 mM NaF, 1 mM Na_3_VO_4_, 100 mg/ml PMSF, and a protease inhibitor cocktail (Roche; Indianapolis, IN, USA)) was used. Proteins were resolved by SDS-PAGE after adjustment of the protein concentration, and subjected to immunoblot analysis with appropriate antibodies as follows: TSG6 (R&D systems, MAB2104; Minneapolis, USA); CD44 (Santa Cruz Biotechnology, sc-9960); PLK (Santa Cruz Biotechnology, sc-17783); phospho-PLK1^T210^ (Cell Signaling, 5472; Danvers, USA); N-cadherin (Sigma, C3865); E-cadherin (Cell Signaling, 4065); Smad2/3 (Cell Signaling, 8685); p-Smad2^S465/S467^ (Cell Signaling, 18338); β-actin (Santa Cruz Biotechnology, sc-47772); GAPDH (Santa Cruz Biotechnology, sc-47724); and IgG (Santa Cruz Biotechnology, sc-2027). Immune complexes were detected using an Odyssey infrared imaging system (LI-COR Biosciences; Lincoln, NE, USA). The values of intensity were determined using Photoshop software.

### Quantitative real time polymerase chain reaction (qRT-PCR)

Total RNA was extracted from cells at 48 h after exposure to TGF-β and quantified by Nanodrop (Thermo Fisher Scientific, Waltham, MA, USA). Next, cDNA was generated by using a First Strand cDNA Synthesis Kit (Thermo Fisher Scientific). The synthesized cDNA was mixed with SYBR Green Master Mix (Bio-Rad Laboratories, Hercules, CA, USA) and gene-specific primers, and then qRT-PCR was conducted using a CFX96 Real-Time PCR system (Bio-Rad Laboratories). The levels of mRNA were normalized using GAPDH as an internal control and all normalized values within the dataset were calculated relative to untreated control cells with the 2^-ΔΔCT^ method 26. The primer sequences used are shown in Table S2.

### Lentivirus-based plasmid expression

For the lentiviral expression of TNFAIP6 (gene ID no. 7130) or PLK1 (gene ID no. 18817), we used pLVX-TRE3G-eRFP and pLVX-Tet3G vectors (Clontech #631351; CA, USA). TNFAIP6 and various versions of PLK1, including wild-type, T210D, and W414F/V415A mutants, were as described previously 12. The various versions of PLK1 were subcloned into pLVX-TRE3G-eRFP and a lentivirus expressing PLK1 was generated according to the manufacturer's guide and previous reports 20. For viral infection, cells were treated with viral particles from pLVX-Tet3G and pLVX-TRE3G-eRFP-PLK1 or pLVX-TRE3G-eRFP-TNFAIP6 with 10 µg/ml polybrene and 10 mM HEPES. G418 and puromycin were treated to select the infected cells at a concentration of 500 µg/ml or and 2 µg/ml for 5 days or 2 days, respectively. The expression of PLK1 or TSG6 was induced with 2 µg/ml of doxycycline.

### Lentivirus-based shRNA preparation

To deplete *TNFAIP6*, we designed lentivirus-based shRNA transfer plasmids to target human *TNFAIP6* (gene access no. NM_007115) at positions 530-550 (CAAATGAGTACGAAGATAA, shTNFAIP6#1) or positions 693-713 (GGGAAGATACTGTGGAGATGA, shTNFAIP6#2), and then the lentivirus was generated. The infected cells were selected using 2 µg/ml puromycin for 2 days.

### Immunohistochemistry analyses from animal studies

Four-week-old BALB/c nude mice (male, Orient Bio Inc., Seoul, Korea) were injected with 1 × 10^6^ A549 cells (in 100 μl PBS) expressing pLVX-TRE3G-eRFP-Tet3G-Mock, wild-type PLK1 (PLK1-WT), T210D mutant PLK1 (PLK1-TD), and W414F/V415A mutant PLK1 (PLK1-FA) through the tail vein as described previously 12. To induce PLK1, doxycycline was administrated in their drinking water at a concentration of 1 mg/ml. All animal experiments were approved and managed by the guidelines of the Institutional Animal Care and Use Committee, Hanyang University (HY-IACUC-2017-0115A), as described previously 12. Twelve weeks after the injection, mice were sacrificed. The lungs were separated and fixed in 4% paraformaldehyde for the staining of TSG6, CD44, CD68, and CD163. Briefly, tumor tissue sections were deparaffinized and rehydrated with xylene and graded alcohol, respectively. The sections were permeabilized with PBS containing 0.1% Triton X-100 (PBST) at room temperature (RT) and treated with peroxide blocking solution at RT. After washing two times with PBS, the sections were incubated with anti-TSG6 (R&D systems, MAB2104, 1:200), anti-CD44 (Santa Cruz Biotechnology, sc-9960, 1:200), anti-CD68 (Santa Cruz Biotechnology Inc. sc-20060, 1:200), and anti-CD163 (ABclonal Inc., Gyeonggi, Korea, A8383, 1:200) antibodies overnight at 4℃. The next day, the slides were washed three times with Tris-buffered saline and incubated with secondary antibody-conjugated peroxidase. After further washing, immunoperoxidase staining was developed using a DAB chromogen kit (K5007, Dako, Glostrup, Denmark) and counterstained with Meyer's hematoxylin (SH-002, KP&T, Osong, Korea). Images of cells were obtained and evaluated with a confocal microscope FW3000 (Olympus; Tokyo, Japan).

### Immunoprecipitation assay

Cell lysates were incubated with anti-CD44 (Santa Cruz Biotechnology, sc-9960) and normal IgG (Santa Cruz Biotechnology) antibodies for 16 h at 4 °C with end-over-end mixing, followed by incubation with protein G agarose (Santa Cruz Biotechnology) for 4 h at 4°C. Immunoprecipitants were divided from the supernatants by centrifugation and washed four times with lysis buffer. Proteins were resolved by SDS-PAGE and then analyzed by immunoblotting.

### Chromatin immunoprecipitation assays

We examined the interaction between c-Jun (AP-1) and the promoters of *IL6, TNFAIP6*, and *VEGFA* in A549 cells treated with rhTSG6. Cross-linking reaction was performed with 1.4% formaldehyde. Cells were lysed with IP buffer. Chromatin was sheared by sonication and incubated with antibody to c-Jun (Santa Cruz Biotechnology, sc-74543) or normal IgG for 16 hours. Sheared chromatin was incubated with a specific protein A/G bead for 4 hours and then washed five times with IP buffer. To the washed beads, Chelex 100 slurry (Bio-Rad; Hercules, CA, USA) was added and then the samples were boiled and incubated with Proteinase K (Invitrogen; Carlsbad, CA, USA) at 55°C for 30 min. The samples cleared by centrifugation, and the supernatants were taken for real-time PCR. The bound chromatin fractions were amplified with promoter-specific primers of human *IL6, TNFAIP6*, and *VEGFA* (Table S3) for 40 cycles. For the interaction between Smad2/3 and the promoters of *CD274* (encoding PD-L1), *SNAI1*, and *SNAI2* in rhTSG6-treated A549 cells, anti-Smad2/3 antibody (#8685, Cell signaling) and promoter-specific primers of human *CD274, SNAI1*, and *SNAI2* (Table S3) were used. Real-time PCR was performed using SYBR Green Master Mix (Bio-Rad, #1708880) with a CFX96 Real-Time PCR system (Bio-Rad). The data were analyzed using the comparative C_T_ (ΔΔC_T_) method.

### Statistical analysis

All data are expressed as the means ± SDs of at least three independent experiments, each conducted in triplicate. Statistical analysis was performed using the Student's *t*-test and statistical significance was set at *p* < 0.05.

## Results

### Concurrent expression of *TNFAIP6* and anti-inflammatory cytokines correlate with the low survival rate of lung adenocarcinoma patients

**Figure 1:**
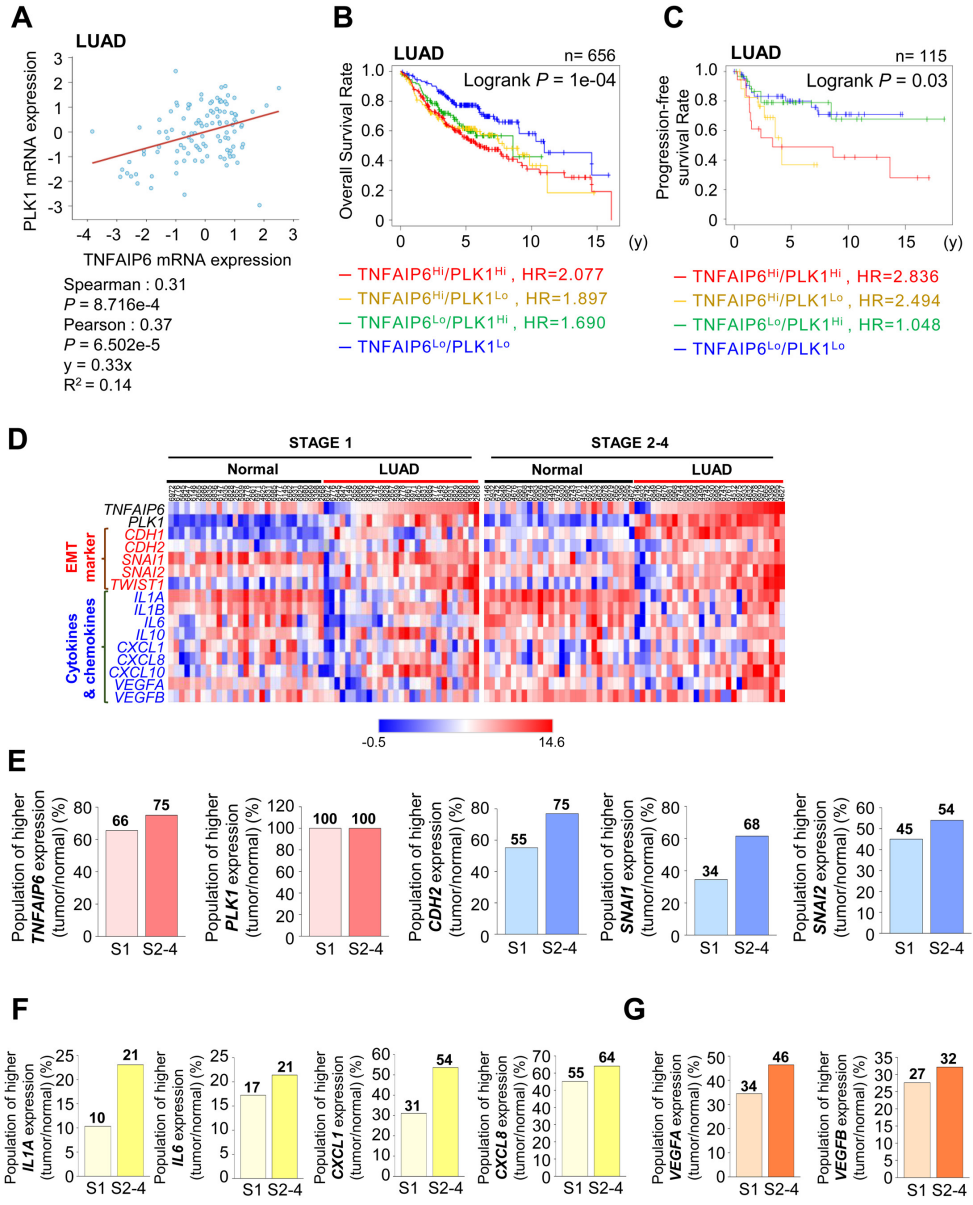
** Clinical correlation between the survival rate of patients and the expression of TSG6 and PLK1 in lung adenocarcinoma (LUAD). (A)** Analysis of Spearman's and Pearson's correlation coefficients between the expression of *TNFAIP6* and *PLK1* in LUAD patients using cBioportal. **(B-C)** The overall survival (OS) of LUAD patients (n = 656, Log-rank *P* = 1e-04) **(B)** and the progression-free survival (PFS) of LUAD patients (n = 115, Log-rank *P* = 0.03) **(C)** were analyzed according to the expression of *TNFAIP6* and *PLK1* using KM PLOTTER. High (Hi) and low (Lo) were generated by dividing patients according to their expression at the median cut-off. **(D)** A heatmap analysis was performed using a dataset of LUAD patients from TCGA for the expression of *TNFAIP6*, *PLK1*, epithelial-mesenchymal transition markers (EMT), and cytokines and chemokines markers in paired normal (left) and tumor tissues (right) depending on stages. **(E)** The ratio of increased expression of *TNFAIP6*, *PLK1*, and EMT markers (*CDH2*, *SNAI1*, and* SNAI2*) and **(F-G)** cytokine and chemokine markers (*IL1A*, *IL6*, *CXCL1*, *CXCL8*, *VEGFA*, and *VEGFB*) in tumor tissue compared to adjacent normal tissue were plotted.

*TNFAIP6* was identified as the most highly expressed gene during active PLK1-driven EMT of NSCLC 12 (Fig. S1A). Because of this, we investigated the correlation between *TNFAIP6* and *PLK1* expression in LUAD and squamous cell carcinoma (LUSQ) (Fig.1A, Fig. S1B). In LUAD, a positive correlation was observed between *TNFAIP6* and *PLK1* mRNA expression (Spearman: 0.31,* p*=8.716e-4; Pearson: 0.37, *p*=6.502e-5) (Fig. 1A). In contrast, a negative correlation was identified in LUSQ (Spearman: -0.43,* p*=1.539e-4; Pearson: -0.37, *p*=1.484e-3) (Fig. S1B). A clinical relevance between the expression of *TNFAIP6*/*PLK1* and overall survival rate (OS) was observed in patients with lung cancer (Fig. S1C), LUAD (Fig. 1B), and LUSQ (Fig. S1D) using a Kaplan-Meier (KM) plot database 22. The OS of LUAD patients (n = 656) with high *TNFAIP6^Hi^/PLK1^Hi^* expression was significantly shorter than those with low *TNFAIP6^Lo^/PLK1^Lo^* expression (red line, HR=2.077, Log-rank *p* =0.0001) (Fig. 1B, Table S1). Additionally, the progression-free survival rate (PFS) of LUAD patients (n = 115) with *TNFAIP6^Hi^/PLK1^Hi^* expression was much shorter than those with low *TNFAIP6^Lo^/PLK1^Lo^* expression (red line, HR=2.836, Log-rank *p* =0.03) (Fig. 1C). However, in LUSQ patients (n = 300), no significant correlation was observed between *TNFAIP6* and *PLK1* expression and OS (Fig. S1D, Table S1). Therefore, the high expression of TNFAIP6 and PLK1 is associated with poor prognosis in LUAD patients, but not in LUSQ.

We then determined the correlation of expression among *TNFAIP6, PLK1,* and mesenchymal markers depending on stages. A heatmap analysis was conducted using a TCGA dataset of LUAD patients for *TNFAIP6, PLK1,* and mesenchymal markers including *CDH2, SNAI1,* and* SNAI2* in paired normal tissue (Fig. 1D-E, left, normal) and adjacent tumor tissues (Fig. 1D-E, right, LUAD) depending on stages. The expression levels and frequencies of *TNFAIP6, PLK1, CDH2, SNAI1,* and* SNAI2* were higher in stages 2-4 than in those of stage 1 (Fig. 1D-E). In the genomic analysis of LUAD, the levels of *TNFAIP6* were higher in patients with advanced stages 2-4 (75%) than in those with tumor stage 1 (66%) (Fig. 1D-E), indicating that *TNFAIP6* and *PLK1* were markedly expressed in advanced LUAD. Previously we found that the immune system process and inflammatory response were ranked within the top 5 pathways in KEGG pathway analysis using invasive cells expressing active PLK1 12. Given that *TNFAIP6* is related to inflammation and cancer 9, the expression of cytokines and chemokines was observed in both normal tissues and adjacent tumor tissues of LUAD patients. The levels of *IL1A* and* IL6* were higher in patients with advanced stages than in those with tumor stage 1 (Fig. 1D-F). In addition, the levels of *CXCL1, CXCL8, VEGFA*, and *VEGFB* related to monocyte recruitment and angiogenesis were higher in patients with advanced stages than in those with tumor stage 1 (Fig. 1D-G). The expression of these cytokines correlated with the expression of *TNFAIP6* in advanced LUAD. Therefore, advanced LUAD showed a higher expression of *TNFAIP6, PLK1,* and cytokines related to monocyte recruitment, macrophage polarization, and angiogenesis.

### TSG6 and its receptor CD44 are highly expressed in TGF-β-induced EMT of LUAD cells

**Figure 2:**
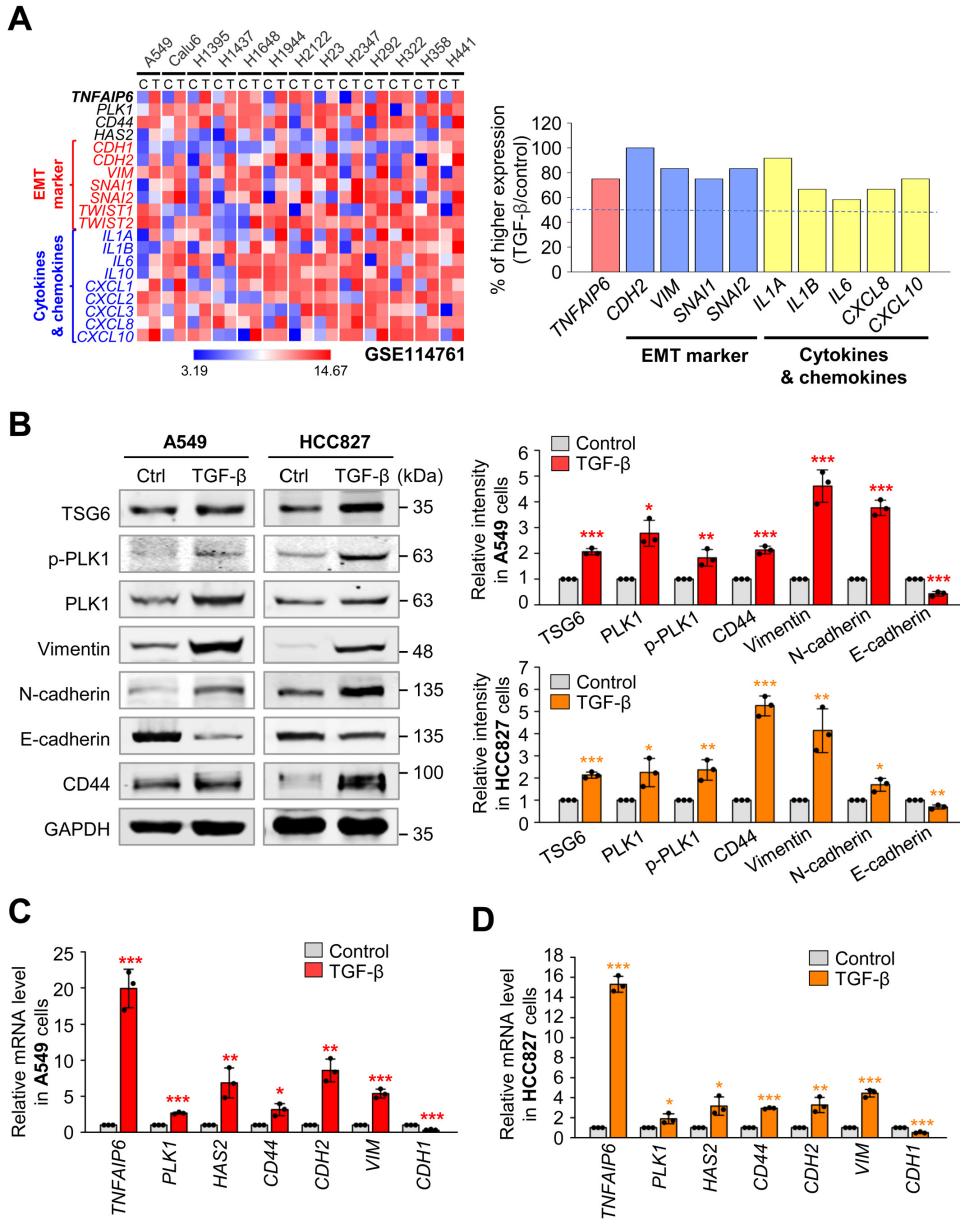
** Co-expression of TSG6 and PLK1 in TGF-β induced EMT of LUAD cells. (A)** The gene expression in the GSE114761 dataset in which EMT was induced by treating the LUAD cell line with TGF-β was visualized as a heatmap (left panel), and the ratio of increased expression of each factor in the TGF-β-treated group compared to the control group was plotted (right panel). **(B-D)** A549 and HCC827 LUAD cells were treated with 2.5 ng/ml of TGF-β for 48 hours. **(B)** Immunoblotting was performed to measure the protein levels of TSG6, p-PLK1, PLK1, Vimentin, N-cadherin, E-cadherin, CD44, and GAPDH in A549 (left panel) and HCC827 (right panel) cells. The relative band intensities were analyzed and plotted in A549 (right upper panel) and HCC827 (right lower panel) cells. *p < 0.05; ** p < 0.01; *** p < 0.001. (n = 3). **(C-D)** *TNFAIP6*, *PLK1*,* HAS2*, *CD44*, *CDH2*, *VIM*, and *CDH1* expression was measured in in A549 **(C)** and HCC827** (D)** cells by qRT-PCR. *p < 0.05; ** p < 0.01; *** p < 0.001. (n = 3).

To investigate the expression of *TNFAIP6* (which encodes TSG6) during LUAD metastasis, we analyzed data from the published transcriptome (GSE114761) of TGF-β-treated LUAD cells including A549, Calu6, NCI-H1395, NCI-H1437, NCI-H1648, NCI-H1944, NCI-2122, NCI-H23, NCI-H2347, NCI-H292, NCI-H322, NCI-H358, and NCI-H441 (Fig. 2A). Heatmap analysis revealed that *TNFAIP6* expression after treatment with TGF-β was significantly higher in approximately 80% of TGF-β-treated LUAD cells compared to the control, when the mesenchymal markers *CDH2* and *VIM* were high, indicating that *TNFAIP6* was upregulated in TGF-β-induced EMT cells. In addition, the levels of its receptor CD44 27 were upregulated in TGF-β-treated LUAD cells.

To confirm these observations, primary LUAD A549 and HCC827 cells were treated with TGF-β to induce the EMT and analyze the levels of TSG6 and CD44 (Fig. 2B-D). As expected, TGF-β treatment reduced the protein and mRNA levels of E-cadherin (encoded by *CDH1*), whereas it increased the levels of mesenchymal markers N-cadherin (encoded by *CDH2*) and vimentin (encoded by *VIM*). Under these conditions, immunoblotting revealed that the levels of TSG6 and CD44 were upregulated in TGF-β-treated A549 and HCC827 cells (Fig. 2B). Furthermore, qRT-PCR analysis showed that *TNFAIP6* expression increased by approximately 15~20-fold in TGF-β-treated A549 and HCC827 cells (Fig. 2C-D). The changes in *CD44*, *CDH2*, *VIM*, and *PLK1* in TGF-β-treated NSCLC cells, were consistent with the data from the transcriptome (GSE114761) of the TGF-β-treated cells. Thus, TSG6 and CD44 were upregulated during TGF-β-induced EMT in LUAD cells.

### Inhibition of PLK1 activity reduces the expression of TSG6 and CD44 in the tail-vein injection mouse model

**Figure 3:**
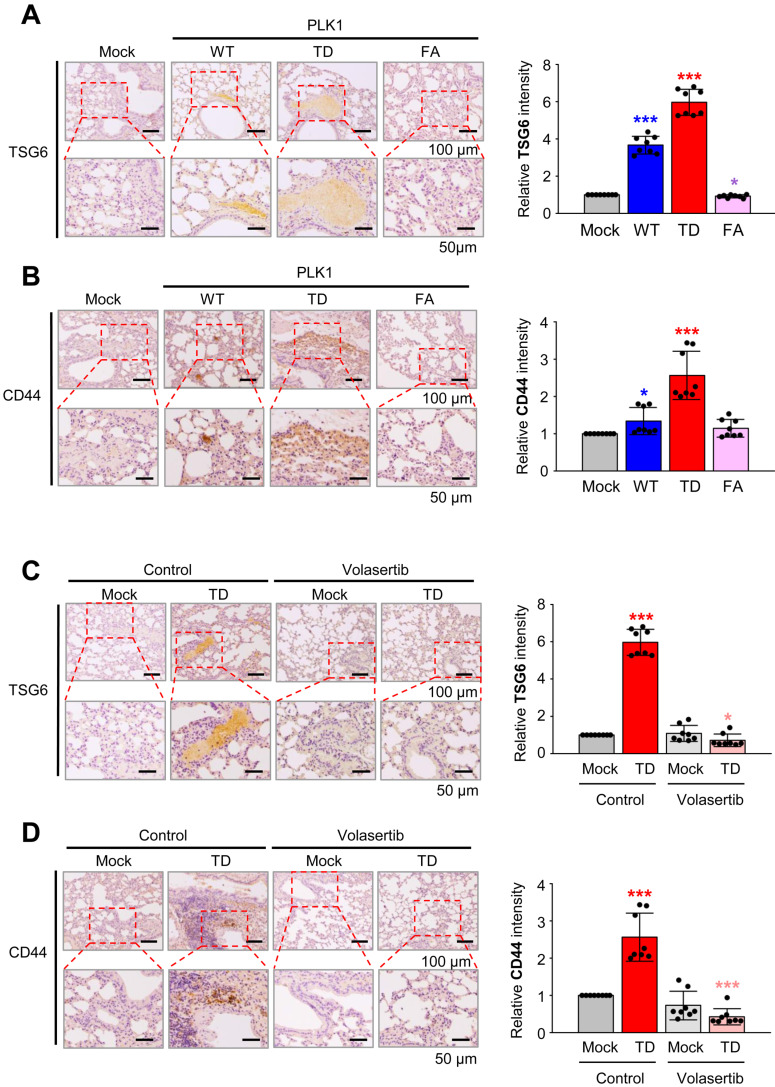
** Expression of TSG6 in an active PLK1-driven metastatic lung cancer mouse model. (A)** Representative TSG6 staining was performed using lung tissue from mice transplanted with wild-type (WT), a constitutively active T210D (TD), and a substrate-nonbinding mutant at W414F/V415A (FA) of the polo-box domain of PLK1 (left panel). The relative density of TSG6 staining was analyzed and plotted (right panel). *p < 0.05; ** p < 0.01; *** p < 0.001. (n = 8).** (B)** Representative CD44 staining was performed using lung tissue from mice transplanted by various versions of PLK1 (left panel), and the relative density of CD44 staining was analyzed and plotted (right panel). *p < 0.05; ** p < 0.01; *** p < 0.001. (n = 8).** (C)** Representative TSG6 staining was performed on the lung tissue of mice transplanted with active PLK1-treated volasertib (PLK1 inhibitor) (left panel), and the relative density of TSG6 staining was analyzed and plotted (right panel). *p < 0.05; ** p < 0.01; *** p < 0.001. (n = 8).** (D)** Representative CD44 staining was performed using lung tissue from mice transplanted with active PLK1-treated volasertib (PLK1 inhibitor) (left panel), and the relative density of CD44 staining was analyzed and plotted (right panel). *p < 0.05; ** p < 0.01; *** p < 0.001. (n = 8).

To explore the role of PLK1 in regulating TSG6 and CD44 expression, we conducted immunohistochemistry on lung tissues of BALB/c nude mice that were injected via the tail vein with A549 cells expressing wild-type PLK1 (PLK1-WT), a constitutively active PLK1 (PLK1-TD), or an inactive mutant of PLK1 (PLK1-FA) 12. The expression of TSG6 and CD44 were higher in tissues of mice injected with A549 cells expressing PLK1-TD compared to those with PLK1-WT or PLK1-FA (Fig. 3A-B). Specifically, the relative intensity of TSG6 (Fig. 3A) or CD44 (Fig. 3B) increased by up to 6.0 or 2.5-fold, respectively, in lung tissues with PLK1-TD. In contrast, TSG6 or CD44 levels decreased by approximately 0.9 or 1.2-fold in tissues from mice injected with A549 cells expressing PLK1-FA compared to that of the mock (Fig. 3A-B). Additionally, treatment with a PLK1 inhibitor volasertib reduced TSG6 or CD44 expression. In this experiment, two weeks after mice were injected in the tail vein with A549 cells expressing PLK1-TD, they were treated intravenously with 20 mg/kg of volasertib weekly for three weeks 12. After 10 weeks, the lung tissues were stained with antibodies against TSG6 or CD44. As expected, volasertib treatment markedly reduced the relative intensity of TSG6 (Fig. 3C) and CD44 (Fig. 3D), even in mice expressing PLK1-TD (Fig. 3C-D). Thus, the expression of TSG6 and CD44 were regulated by PLK1 activity and can be effectively reduced by PLK1 inhibition.

### TSG6 functions as an inducer of EMT by activation of TGF-β/Smad signaling

**Figure 4:**
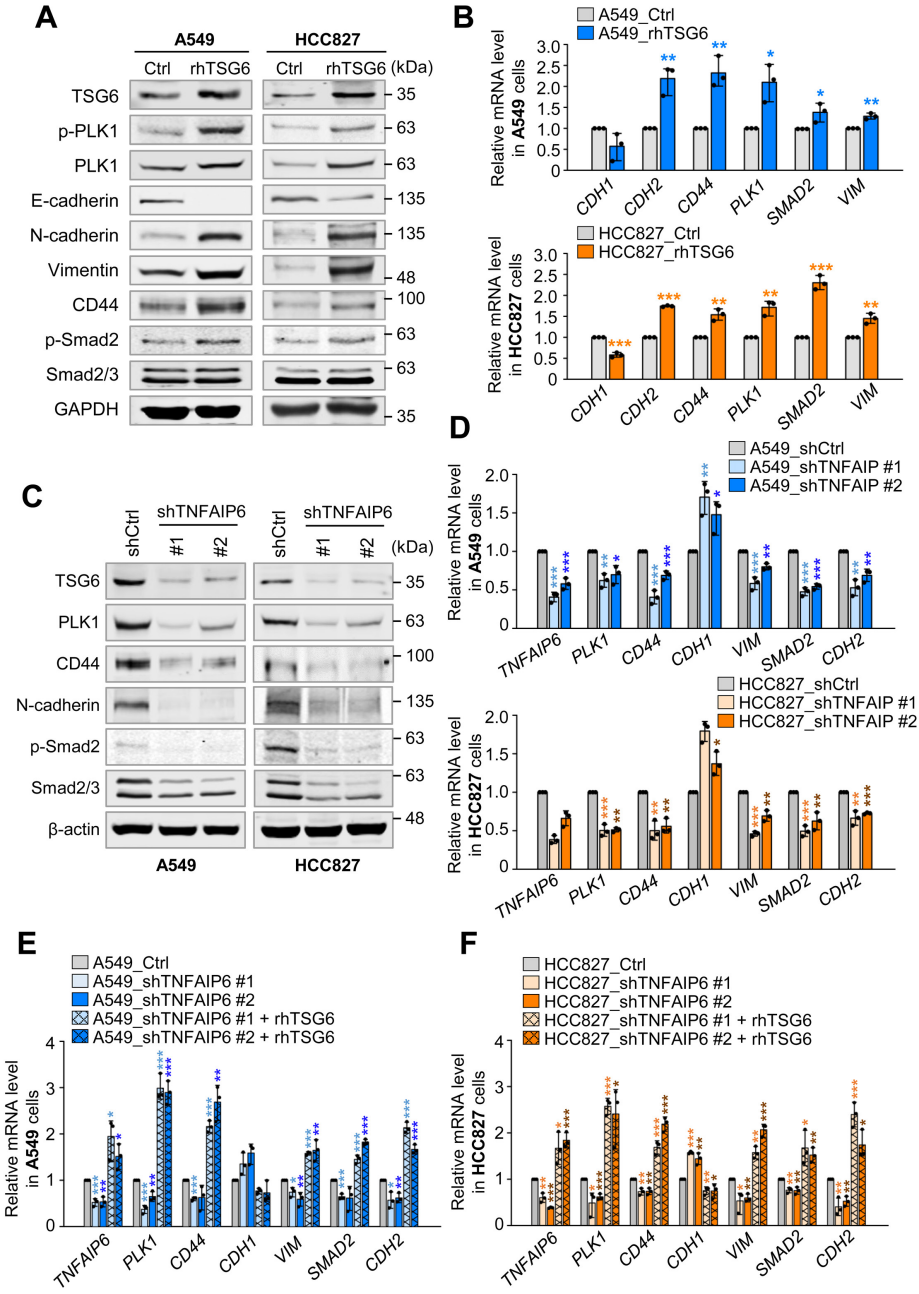
** Recombinant TSG6 protein induced EMT by activation of TGF-β signaling. (A-B)** A549 and HCC827 cells were treated with 200 ng/ml of rhTSG6 for 2 hours. **(A)** Immunoblotting was performed to measure the protein levels of TSG6, PLK1, p-PLK1^T210^, E-cadherin, N-cadherin, Vimentin, CD44, p-Smad2^S465/S467^, Smad2 and GAPDH in A549 (left panel) and HCC827 (right panel) cells. **(B)** qRT-PCR was used to evaluate *CDH1*, *CDH2*,* CD44*,* PLK1*,* SMAD2*, and *VIM* expression in A549 (right upper panel) and HCC827 (right lower panel) cells. *p < 0.05; ** p < 0.01; *** p < 0.001. (n = 3). **(C-F)** TSG6 shRNA targeting the positions 530-550 (#1) or 693-713 (#2) was applied to A549 and HCC827 cells. **(C)** Immunoblotting was performed to measure the protein levels of TSG6, PLK1, CD44, N-cadherin, Smad2/3, p-Smad2^S465/S467^, and β-actin in A549 (left panel) and HCC827 (right panel) cells. **(D)** qRT-PCR was used to evaluate *TNFAIP6*, *PLK1*,* CD44*,* CDH1*,* VIM*, *SMAD2*, and *CDH2* expression in A549 (upper panel) and HCC827 (lower panel) cells. *p < 0.05; ** p < 0.01; *** p < 0.001. (n = 3). **(E-F)** qRT-PCR was used to evaluate *TNFAIP6*, *PLK1*,* CD44*,* CDH1*,* VIM*, *SMAD2*, and *CDH2* expression in A549** (E)** and HCC827 **(F)** cells after rhTSG6 treatment. *p < 0.05; ** p < 0.01; *** p < 0.001. (n = 3).

Given that TSG6 was highly upregulated in TGF-β-induced EMT (Fig. 2) and was the most highly expressed gene in active PLK1-driven EMT of NSCLC cells (Fig. S1A), we investigated whether TSG6 plays a role in inducing EMT in LUAD cells (Fig. 4). To accomplish this, the levels of epithelial and mesenchymal markers were observed by immunoblotting in recombinant human TSG6 (rhTSG6) protein-treated A549 and HCC827 cells (Fig. 4A). The rhTSG6 treatment increased the levels of vimentin and N-cadherin while decreasing the epithelial marker E-cadherin in both cell lines (Fig. 4A). At the same time, the levels of total and its phosphorylated form at T210 of PLK1 were higher than those of control cells (Fig. 4A). In addition, the levels of its receptor CD44 were upregulated in rhTSG6-treated LUAD cells. To understand the signaling pathway for triggering EMT, the levels of Smad2 and p-Smad2 were observed (Fig. 4A). The data showed that the levels of Smad2 and p-Smad2 were higher than those of the control in rhTSG6-treated cells, indicating that TSG6 induces EMT via TGF-β signaling (Fig. 4A). Consistent with these findings, rhTSG6 treatment also increased the mRNA levels of mesenchymal markers (e.g. *CDH2* and *VIM*), *PLK1*, and *CD44* (Fig. 4B).

Next, we investigate the loss of function effects of TSG6 using shRNA targeting *TNFAIP6* at positions 530-550 (shTNFAIP6#1) or positions 693-713 (shTNFAIP6#2) in A549 and HCC827 cells (Fig. 4C-D). Depletion of TSG6 reduced the levels of N-cadherin and upregulated the levels of E-cadherin under low levels of TSG6 protein (Fig. 4C, Fig. S2A-B). In addition, the protein levels of CD44, PLK1, Smad2/3, and phosphorylated Smad2 were downregulated by the depletion of TSG6. These changes were similar at the mRNA level. When TSG6 was depleted, the expression of mesenchymal markers, *CD44*, and *PLK1* were downregulated (Fig. 4D). These results indicate that TSG6 is important to induce EMT by activating TGF-β signaling, involving its receptor CD44. To understand the importance of TSG6 in EMT, we performed a rescue experiment in TSG6-depleted cells by treating with rhTSG6 protein (Fig. 4E-F). The levels of *TNFAIP6* mRNA reduced by treatment with shRNA (#1 and #2), which was reversed by treatment with rhTSG6 in A549 and HCC827 cells. Under the conditions, the downregulated levels of *CDH2*, *VIM*, *SMAD2*, *CD44*, and *PLK1* caused by *TNFAIP6* knockdown were restored by rhTSG6 treatment. These data indicate that TSG6 is crucial for inducing EMT by the activation of TGF-β signaling.

### TSG6 induces the polarization of tumor-associated macrophages in LUAD microenvironment

In LUAD with high levels of *TNFAIP6* and *PLK1*, cytokines and chemokines associated with inflammation were markedly upregulated compared to normal tissues (Fig. 1D). Furthermore, microarray analysis of PLK1-TD expressing cells identified that the immune system process plays a key role in the tumor microenvironment (Fig. S3A). To explore whether TSG6 affects the tumor microenvironment, lung fibroblast MRC-5 cells were treated with rhTSG6 (Fig. S3B). Markers for cancer-associated fibroblast (CAF) such as PDGFR, FAP1, and SMA 28 were observed after treatment. However, these markers showed modest changes compared to the control, suggesting that TSG6 does not significantly alter CAF activation in the LUAD TME.

**Figure 5:**
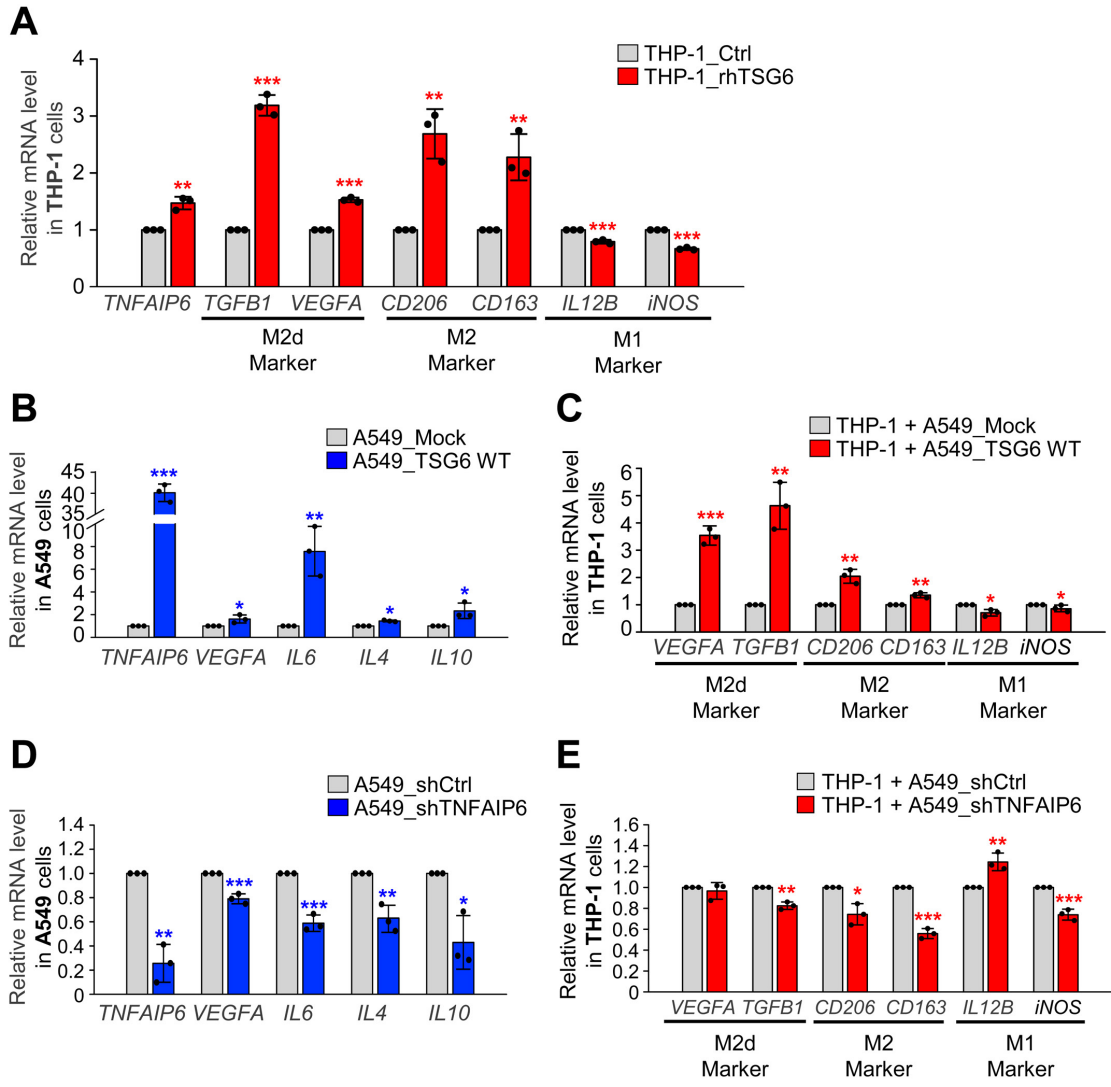
** TSG6 induced the polarization of tumor-associated macrophages. (A)** Monocyte THP-1 cells were treated with 200 ng/ml of rhTSG6 for 8 hours, and qRT-PCR was used to measure *TNFAIP6*, *TGFB1*, *VEGFA* (M2d macrophage markers), *CD206*, *CD163* (M2 macrophage markers), *IL12B*, and *iNOS* (M1 macrophage markers) expression. **(B-C)** TSG6- overexpressing A549 cells and monocyte THP-1 cells were cocultured. **(B)** qRT-PCR was used to evaluate *TNFAIP6*, *VEGFA*,* IL6*,* IL4* and *IL10* expression in A549 cells. *p < 0.05; ** p < 0.01; *** p < 0.001. (n = 3). **(C)** qRT-PCR was used to evaluate *VEGFA*, *TGFB1* (M2d macrophage markers), *CD206*, *CD163* (M2 macrophage markers), *IL12B*, and *iNOS* (M1 macrophage markers) expression in THP-1 cells. *p < 0.05; ** p < 0.01; *** p < 0.001. (n = 3). **(D-E)** TSG6-depleted A549 cells and monocyte THP-1 cells were cocultured. **(D)** qRT-PCR was used to evaluate *TNFAIP6*, VEGFA,* IL6*,* IL4* and *IL10* expression in A549 cells. *p < 0.05; ** p < 0.01; *** p < 0.001. (n = 3). **(E)** qRT-PCR was used to evaluate *VEGFA*, *TGFB1*, *CD206*, *CD163*, *IL12B*, and *iNOS* expression in THP-1 cells. *p < 0.05; ** p < 0.01; *** p < 0.001. (n = 3).

Tumor-secreted factors are known to drive the polarization of M2d-TAM in human monocyte THP-1 cells, particularly through the elevated expression of cytokines in invasive tumor cells 2, 4. To understand whether TSG6 contributes to macrophage polarization in the TME, we treated human monocyte THP-1 cells with rhTSG6 and assessed marker expression using qRT-PCR (Fig. 5A). Treatment with rhTSG6 led to a slight downregulation of M1 macrophage markers *IL12B* and *iNOS*. Conversely, M2 macrophage markers *CD163* and* CD206* were slightly upregulated with their expression increasing by 2.2 and 2.8-fold, respectively, compared to the control. Furthermore, the mRNA levels of TGF-β and VEGFA, key markers of TAMs in the TME, were also elevated following rhTSG6 treatment (Fig. 5A). These data indicate that TSG6 promotes the TAM polarization of monocytes, contributing to the immune modulation of the LUAD microenvironment.

To clarify the role of TSG6 on TAM polarization within the TME, TSG6 was expressed in A549 cells using a doxycycline-inducible expression system (Fig. 5B). A549 cells expressing TSG6 were cocultured with THP-1 monocytes (Fig. 5B-C). When *TNFAIP6* mRNA expression in A549 cells increased by up to 40-fold, the expression of *VEGFA* or *IL6* was also upregulated. In the THP-1 cells cocultured with TSG6-expressing A549 cells, M2 and M2d macrophage markers such as *VEGFA, TGFB1, CD206*, and *CD163* were markedly increased. Conversely, M1 makers *IL12B* and* iNOS* were downregulated compared to THP-1 cells cocultured with the mock A549 cells (Fig. 5B-C), indicating that TSG6 secretion from LUAD cells promotes TAM polarization in the surrounding microenvironment.

To confirm this, TSG6 was depleted using shRNA targeting *TNFAIP6* in A549, and THP-1 monocytes were cocultured with TSG6-depleted A549 cells (Fig. 5D-E). Depletion of *TNFAIP6* in A549 cells led to the downregulation of *VEGFA* or *IL6*. In THP-1 cells cocultured with TSG6-depleted A549 cells, M2 and M2d markers, including *VEGFA, TGFB1, CD206*, and *CD163*, were markedly reduced (Fig. 5D). In contrast, the levels of M1 marker *IL12B* in THP-1 cells cocultured with *TNFAIP6*-depleted A549 cells were higher than those with control shRNA-depleted A549 cells (Fig. 5E). These findings suggest that TSG6 promotes TAM polarization of monocytes, playing a crucial role in modulating the immune microenvironment in LUAD.

### TAM polarization was observed in a tail-vein metastasis mouse model driven by active PLK1

**Figure 6:**
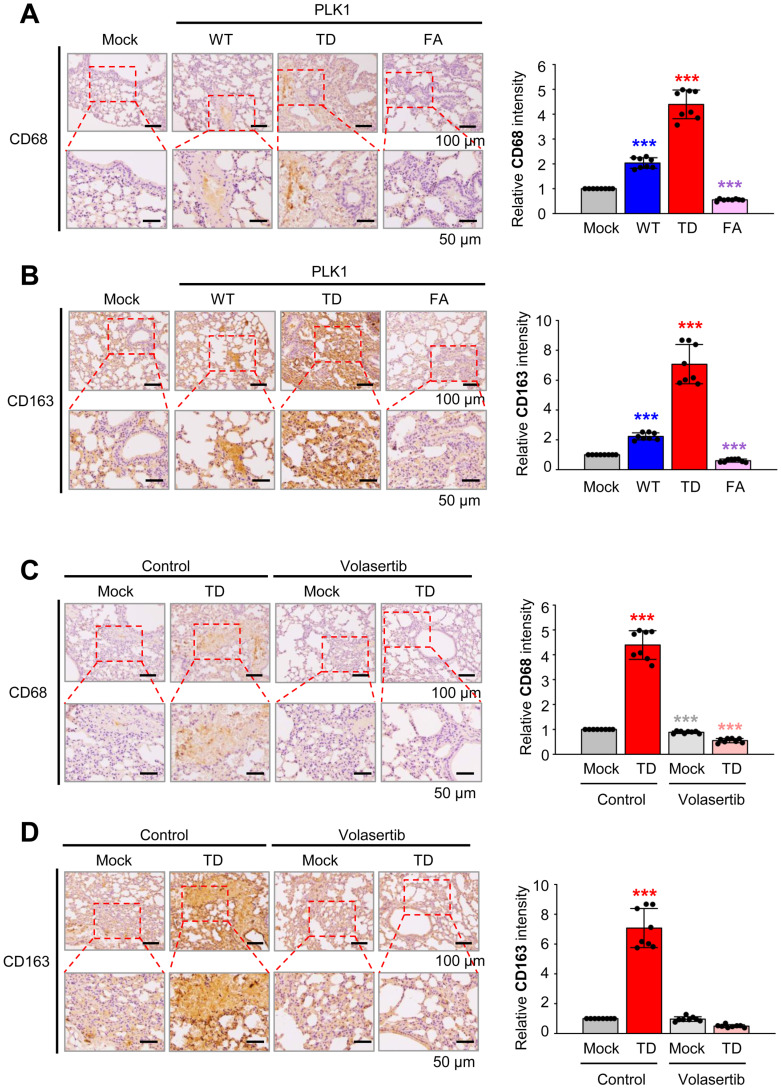
** TAM polarization was induced in active PLK1-driven metastatic lung cancer of a mouse model. (A)** Representative CD68 (pan-macrophage marker) staining was performed on lung tissue of mice transplanted with various versions of PLK1 (left panel), and the relative density of CD68 staining was analyzed and plotted (right panel). *p < 0.05; ** p < 0.01; *** p < 0.001. (n = 8).** (B)** Representative CD163 (M2 macrophage marker) staining was performed on the lung tissue of mice transplanted with various versions of PLK1 (left panel), and the relative density of CD163 staining was analyzed and plotted (right panel). *p < 0.05; ** p < 0.01; *** p < 0.001. (n = 8).** (C)** Representative CD68 staining was performed on the lung tissue of mice transplanted with cells containing active PLK1 treated with volasertib (PLK1 inhibitor) (left panel), and the relative density of CD68 staining was analyzed and plotted (right panel). *p < 0.05; ** p < 0.01; *** p < 0.001. (n = 8).** (D)** Representative CD163 staining was performed using lung tissue from mice transplanted with cells containing active PLK1-treated volasertib (PLK1 inhibitor) (left panel), and the relative density of CD163 staining was analyzed and plotted (right panel). *p < 0.05; ** p < 0.01; *** p < 0.001. (n = 8).

To observe TAM polarization in an *in vivo* model, immunohistochemistry was performed with antibodies against CD163 (a TAM marker) and CD68 (a pan-macrophage marker) on the lung tissue of BALB/c nude mouse injected via the tail vein with A549 cells expressing PLK1-WT, PLK1-TD, or PLK1-FA (Fig. 6A-B), because TSG6 was highly expressed in metastatic tissue nodules expressing PLK-TD (Fig. 3A). The expression of CD68 (Fig. 6A) and CD163 (Fig. 6B) was higher in tissues from mice injected with A549 cells expressing PLK1-TD compared to those injected with PLK1-WT or PLK1-FA. Specifically, the relative intensities of CD68 (Fig. 6A) and CD163 (Fig. 6B) increased approximately 4.5- and 7-fold in lung tissues with PLK1-TD. In contrast, CD68 (Fig. 6A) and CD163 (Fig. 6B) expressions were downregulated approximately 0.5-fold in the tissues of mice injected with A549 cells expressing PLK1-FA compared to those of mock.

Then the expression of CD68 (Fig. 6C) and CD163 (Fig. 6D) were measured when BALB/c nude mice were treated with volasertib, a PLK1 inhibitor (Fig. 6C-D). Volasertib was injected intravenously (at a dose of 20 mg/kg) weekly for three weeks after A549 cells expressing PLK1-TD were injected into mice 12. After 10 weeks, lung tissues were stained with antibodies against CD68 (Fig. 6C) and CD163 (Fig. 6D). The relative intensity of CD68 and CD163 were markedly reduced by approximately 6-fold and 14-fold in volasertib-treated PLK1-TD expressing mice, respectively (Fig. 6C-D). Therefore, TAM polarization is promoted in TSG6-upregulated cells driven by active PLK1 within the tumor microenvironment.

### TSG6 induces the polarization of TAMs through the activation of MAPK/ERK and TGFβR1/Smad signaling pathways

**Figure 7:**
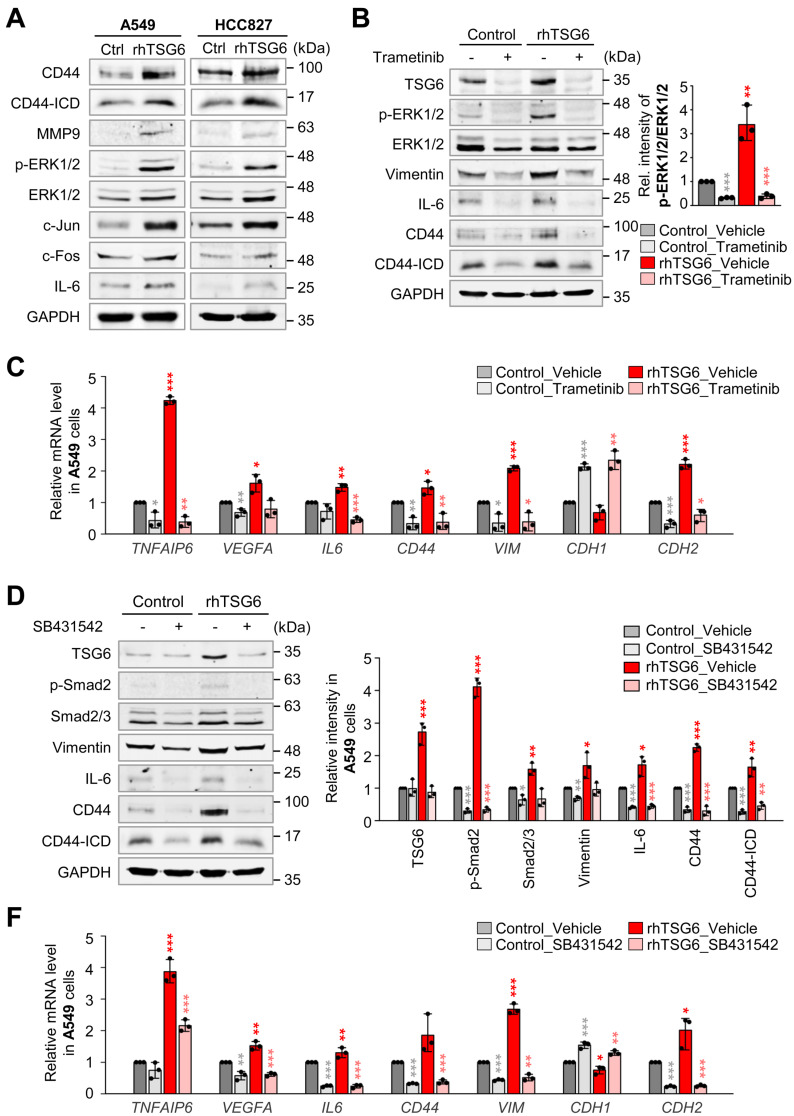
** MAPK/ERK and TGFβR1/Smad signaling are the main pathways for TSG6-mediated EMT and TAM polarization. (A)** A549 and HCC827 cells were treated with 200 ng/ml of rhTSG6 for 2 hours. Immunoblotting was performed to measure the protein levels of CD44, cleaved CD44 (CD44-ICD), MMP9, p-ERK1/2, ERK1/2, c-Jun, c-Fos, IL-6, and GAPDH in A549 (left panel) and HCC827 (right panel) cells. **(B-C)** A549 cells were treated with 10 μM trametinib (MEK1/2 inhibitor) for 48 hours and treated with 200 ng/ml of rhTSG6 for 2 hours. **(B)** Immunoblot was performed to measure the protein levels of TSG6, p-ERK1/2, ERK1/2, vimentin, IL-6, CD44, CD44-ICD, and GAPDH, and the relative band intensity values were analyzed in A549 cells (Sup Fig. S4). *p < 0.05; ** p < 0.01; *** p < 0.001. (n = 3). **(C)** qRT-PCR was used to evaluate *TNFAIP6*, *VEGFA*, *IL6*, *CD44*, *VIM*, *CDH1*, and *CDH2* expression in A549 cells. *p < 0.05; ** p < 0.01; *** p < 0.001. (n = 3). **(D-F)** A549 cells were treated with 30 μM SB431542 (TGFβR1 inhibitor) for 48 hours and with 200 ng/ml of rhTSG6 for 2 hours. **(D)** Immunoblot was performed to measure the protein levels of TSG6, p-Smad2, Smad2/3, vimentin, IL-6, CD44, CD44-ICD, and GAPDH **(D)**, and the relative band intensity values were analyzed in A549 cells **(E)**. *p < 0.05; ** p < 0.01; *** p < 0.001. (n = 3). **(F)** Expression of *TNFAIP6*, *VEGFA*, *IL6*, *CD44*, *VIM*, *CDH1*, and *CDH2* in A549 cells was measured by qRT-PCR. *p < 0.05; ** p < 0.01; *** p < 0.001. (n = 3).

Next, we aimed to explore which signaling pathway is activated by TSG6 to induce TAM polarization and EMT. Previous studies demonstrated that CD44 interacts with EGFR potentiating MAPK/ERK pathway 13, 29 and TGFβR1 30 in human cancer cells. In Figure 4, rhTSG6 treatment induces EMT by the activation of TGF-β signaling. To investigate the activation of the MAPK/ERK pathway by TSG6 treatment, we measured the levels of p-ERK1/2 and AP-1, a complex of c-Jun and c-Fos. We discovered that they were upregulated by TSG6. Simultaneously, the levels of MMP9, IL-6, and total CD44 and the cleaved intracellular domain (ICD) of CD44 (CD44-ICD) were upregulated in rhTSG6-treated LUAD cells (Fig. 7A, Fig. S4A-B).

To further dissect the signaling pathway, rhTSG6-treated A549 cells were treated with MEK inhibitor trametinib and TGFβR inhibitor SB431542 (Fig. 7B-F). Trametinib treatment reduced the levels of p-ERK1/2 (normalized to total ERK1/2) by approximately 89% in TSG6-treated cells (vehicle *vs* trametinib, 3.4 *vs* 0.4) (Fig. 7B, right panel). Additionally, the levels of total CD44, cleaved CD44-ICD, and TSG6 that were increased by TSG6 treatment were markedly reduced upon trametinib treatment (Fig. 7B, Fig. S4C). Trametinib also suppressed the expression of vimentin and IL-6, indicating that MEK is important for TSG6-mediated EMT and TAM polarization. Furthermore, trametinib downregulated TSG6-induced mRNA levels of *TNFAIP6* and* CD44* (Fig. 7C). For TAM polarization, the upregulated *VEGFA* and* IL6* mRNAs induced by TSG6 were suppressed in trametinib-treated A549 cells. Similarly, the mesenchymal markers *VIM* and *CDH2* were downregulated following trametinib treatment. Therefore, MAPK/ERK signaling is important for TSG6-mediated EMT and TAM polarization.

To investigate whether TGFβR/Smad signaling is involved in TSG6-mediated EMT, given that TSG6 treatment induced EMT and Smad2 activation (Fig. 4A, C), we examined the effects of TGFβR inhibitor SB431542 on rhTSG6-treated A549 cells (Fig. 7D-F, Fig. S4D). SB431542 treatment reduced the levels of p-Smad2 (normalized to total Smad2) by approximately 83% (vehicle *vs* SB431542: 2.8 *vs* 0.5) in rhTSG6-treated cells (Fig. S4D). Additionally, SB431542 treatment decreased the TSG6-induced upregulation of total CD44, cleaved CD44-ICD, vimentin, and IL-6 at both the protein and mRNA levels (Fig. 7D-F). These findings indicate that TGFβR/Smad signaling is critical for TSG6-mediated EMT and TAM polarization. Collectively, both MAPK/ERK and TGFβR/Smad signaling play pivotal roles in TSG6-mediated EMT and TAM polarization.

### TSG6 promotes the interaction between CD44 and TGFβR or EGFR, driving the expression of genes involved in EMT and TAM polarization

**Figure 8:**
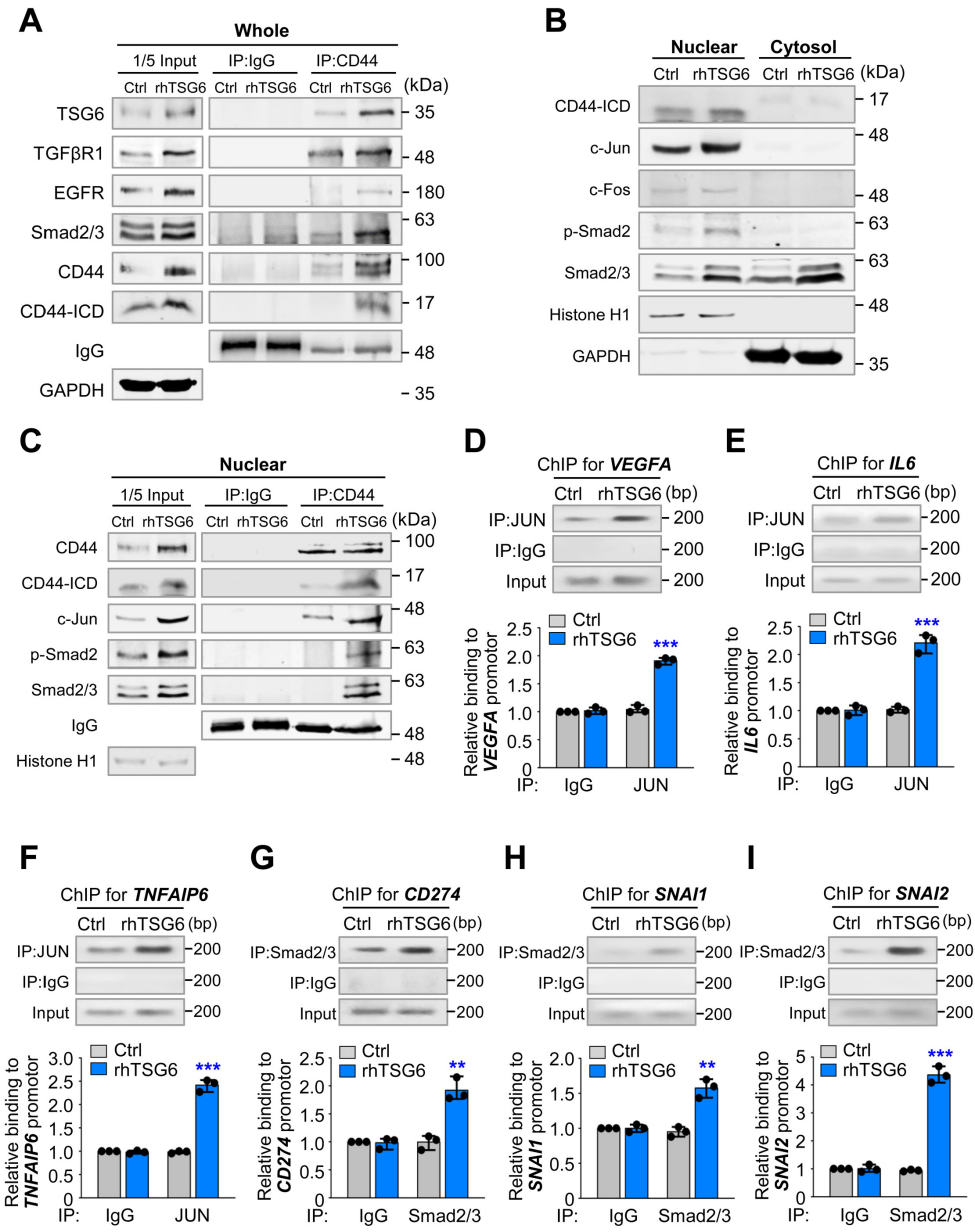
** TSG6 facilitates the interaction between CD44 and TGFβR or EGFR for the expression of genes involved in EMT and TAM polarization. (A)** A549 cells were treated with 200 ng/ml of rhTSG6 for 2 hours. Immunoprecipitation and immunoblotting were used to measure the protein levels of CD44, CD44-ICD, TGFβR1, EGFR, and GAPDH in A549 cells. **(B)** Fractionation of A549 cells treated with rhTSG6 was used to observe the levels of nuclear and cytosolic fraction. Immunoblotting was used to measure the protein levels of CD44, CD44-ICD, c-Jun, c-Fos, Smad2/3, Histone H1 (nuclear loading control), and GAPDH (cytosolic loading control). **(C)** Nuclear fraction of A549 cells treated with 200 ng/ml of rhTSG6 for 2 hours was used for immunoprecipitation and immunoblotting analysis. Immunoblot analyses were performed using anti-CD44, anti-c-Fos, and anti-c-Jun antibodies. **(D-F)** ChIP assays for AP-1 (complex of c-Jun and c-Fos) binding to the promoters of *VEGFA* **(D)***, IL6* **(E)**, and *TNFAIP6* **(F)** in rhTSG6-treated A549 cells. **(G-I)** ChIP assays for Smad2/3 binding to the promoters of *CD274* (G)*, SNAI1* **(H)**, and *SNAI2* **(I)** in rhTSG6-treated A549 cells. Assays were performed on chromatin fragments using antibody to c-Jun **(D-F)** or Smad2/3 **(G-I)** and normalized to pre-immune normal IgG. Immunoprecipitated fractions were assayed by qRT-PCR to determine the binding to each promoter. Data are presented as mean ± SD of three independent experiments (significantly different from the experimental control). *p < 0.05; **p < 0.01; ***p < 0.001; (*n =* 3).

Next, we aimed to investigate whether TSG6 influences the direct interaction between CD44 and TGFβR or EGFR, as MAPK/ERK and TGFβR/Smad signaling were activated in the presence of TSG6 (Fig. 7). Previous studies have reported that CD44 interacts with TGFβR1 30 or EGFR 29 in cancer. To examine this, we conducted an immunoprecipitation assay using anti-CD44 antibody in rhTSG6-treated A549 cells (Fig. 8A). Our results indicated that CD44 interacted with TSG6 abundantly in rhTSG6-treated A549 cells (Fig. 8A). Additionally, TGFβR1 and EGFR were also detected, although the levels of EGFR were lower than those of TGFβR1 in these cells. Given that cleaved CD44-ICD translocates to the nucleus and regulates transcriptional activity 31, we examined its subcellular localization in rhTSG6-treated A549 cells (Fig. 8B). The levels of CD44-ICD were higher in the nucleus compared to the cytosol and TSG6 treatment upregulates its nuclear location. In rhTSG6-treated cells, c-Jun and c-Fos, components of AP-1, which are transcriptional factors downstream of MAPK/ERK1/2 signaling, were upregulated (Fig. 8B). In addition, p-Smad2 was upregulated in the nucleus after TSG6 treatment, indicating that TGFβR1-mediated signaling is activated (Fig. 8B). Furthermore, CD44-ICD was found to interact with c-Jun, in the nuclear fraction of rhTSG6-treated A549 cells (Fig. 8C). These findings suggest that the nuclear translocation of CD44-ICD enables its interaction with AP-1, contributing to transcriptional regulation in the presence of TSG6. Then to determine whether AP-1 transcriptionally activates genes related to TAM polarization, such as IL-6 and VEGFA, a ChIP assay was conducted in rhTSG6-treated cells using anti-c-Jun antibody (Fig. 8D-E). In rhTSG6-treated cells, the expression of* IL6, VEGFA,* and *TNFAIP6* increased approximately a 2.5-fold compared to controls (Fig. 8D-F), suggesting that AP-1 directly bound to the promoters of *IL6, VEGFA,* and *TNFAIP6.* Then to determine whether TGFβR1-mediated signaling is activated for EMT and immune escape by TSG6 treatment, a ChIP assay was performed with anti-Smad2/3 antibody. In rhTSG6-treated A549 cells, the expression of *CD274, SNAI1,* and *SNAI2* increased approximately a 2-, 1.7-, and 4.3-fold increase, respectively, compared to controls, indicating that Smad2/3 directly bound to the promoters of *CD274, SNAI1,* and *SNAI2* for immune escape and EMT (Fig. 8G-I).

In summary, TSG6 treatment promotes interaction between CD44 and TGFβR or EGFR, leading to the activation of TGFβR/Smad or MAPK/ERK signaling pathways and triggering the cleavage of CD44-ICD, which accordingly translocates to the nucleus. In the nucleus, CD44-ICD interacts with transcriptional factors Smad2/3 or AP-1 to induce the expression of genes related to EMT, such as *SNAI1* and *SNAI2*, and TAM polarization, such as *VEGFA* and *IL6*.

## Discussion

Previously, we observed high expression of *TNFAIP6* ranked with top in active PLK1-driven EMT 12. In this study, we addressed the effects of TSG6 on EMT and the tumor microenvironment, especially monocyte polarization. Microarray analysis of invasive NSCLC cells in active PLK1-induced EMT recognized TSG6 as the most highly expressed factor 12, with the immune processes emerging as a prominent biological process in KEGG pathway analysis. In LUAD patients, highly co-expressed TSG6 and PLK1 correlated with lower OS rates and a higher hazard ratio than 1, as evaluated by KM plot analysis. We further found that the expression of TSG6, CD44, CD68, and CD163 (marker of TAMs) is elevated in metastatic lung cancer tissues of mice driven by active PLK1. The upregulations of TSG6, CD44, CD68, and CD163 were suppressed by the treatment with PLK1 inhibitor volasertib (Fig. 3 and 6), indicating that PLK1 activity is important to regulate the expression of TSG6, CD44, CD68, and CD163 for modulating TME. In our previous study, we also found that β-catenin, a transcriptional factor of TSG6 and CD44, is phosphorylated by PLK1, which enhances its transcriptional activity and extracellular remodeling in advanced NSCLC 20. Based on this study, the upregulations of TSG6 and CD44 during EMT would be explained by activation of β-catenin by PLK1-mediated phosphorylation in LUAD. The PLK1 inhibitor volasertib suppresses PLK kinase activity, blocking the phosphorylation of β-catenin and reducing its stability. As a result of β-catenin degradation, the expression of its downstream targets, including TSG6 and CD44, would be decreased in LUAD. Notably, TSG6 also showed as an inducer of EMT in LUAD through TGF-β/Smad signaling in Figure 4. In A549 and HCC827 cells treated with TSG6, mesenchymal markers including vimentin and N-cadherin were upregulated (Fig. 4A-B). TSG6-induced EMT was activated by TGF-β signaling, as evidenced by increased phosphorylation of Smad2 in TSG6-treated LUAD cells. Depletion of TSG6 resulted in the downregulation of mesenchymal markers and p-Smad2 (Fig. 4C-D), which was restored by treatment with rhTSG6 (Fig. 4E-F). These data indicate that TSG6 would be a key driver of EMT in LUAD. Because PLK1 is upregulated and activated during the EMT in NSCLC 12, it is possible that TSG6 and PLK1 are indirectly linked through reciprocal regulation.

The function of TSG6 would be associated with HA and CD44, a receptor of both HA or TSG6 27. As CD44 serves as a receptor for TSG6, we hypothesized that binding TSG6 to CD44 triggers TGF-β/Smad signaling or MAPK kinase signaling through the formation of a complex with TGFβR1 or EGFR. In the results, inhibition of TGFβR1 or MEK1/2 significantly reduced TSG6-induced EMT and cytokine expression related to TAM polarization (Fig. 7). Notably, the direct interaction between CD44 and TGFβR1 or EGFR was increased following rhTSG6 treatment (Fig. 8A). Therefore, TSG6 facilitates the interaction between CD44 and TGFβR1 or EGFR. The results are supported by the previous studies 30 showing that hyaluronan, another ligand of CD44 and an interacting partner with TSG6, promotes signaling interaction between CD44 and TGFβR1 in metastatic breast tumors 30. Additionally, the interaction between CD44 and EGFR has been shown to initiate and drive the progression of head and neck squamous cell carcinoma 13, 29. The cleavage of CD44 may be influenced by its interaction with TGFβR1 or EGFR in the presence of TSG6 by upregulated MMP9. In addition, the upregulation of CD44-ICD was suppressed by treatment with the TGFβR1 inhibitor SB431542 or the MEK inhibitor trametinib. Thus, the increased CD44-ICD, driven by TSG6-CD44-TGFβR1 or EGFR-mediated signaling, translocated into the nucleus, where it contributes to transcriptional regulation via Smad2/3 or AP-1.

TSG6 secretion drives the polarization toward M2d-TAMs from monocytes. In patients with advanced LUAD, the expression levels of mesenchymal markers and cytokines associated with M2d-TAM polarization were significantly higher in LUAD tissues compared to normal tissues (Fig. 1). When rhTSG6 was treated to monocyte cells, the expression of M2d markers increased in THP-1 cells (Fig. 5). Similar increases of M2d inducers were also detected in A549 cells treated with TSG6. These findings demonstrate that TSG6 induces monocyte polarization toward M2d-TAM in the TME. A recent study showed that TSG6 promotes tumor migration and invasion in a colorectal cancer model by reprogramming normal fibroblasts into CAFs 13. In our study, the reprogramming effects of TSG6 on normal lung fibroblasts into CAFs (Fig. S3B) is potentially due to tissue-specific differences. However, we found that TSG6 strongly promotes M2d-TAM polarization in the LUAD tumor microenvironment (Fig. 5-6). This finding is supported by previous studies showing TSG6-mediated M2 macrophage polarization in alcoholic hepatitis *in vivo* 32 and in DSS-induced colitis of mice 33. Thus, the evidence supports our study that TSG6 secreted by LUAD cells drives monocyte polarization into M2d-TAMs within the TME. However, it remains to be determined if TSG6 promotes M2d-TAMs polarization *in vivo* as a further study.

In conclusion, we suggest that TSG6 would be a crucial factor that contributes to tumor malignancy by inducing TAM polarization in TME. TSG6 treatment facilitates the interaction between CD44 and TGFβR or EGFR, which leads to the activation of TGFβR/Smad or MAPK/ERK signaling pathways and cleavage of CD44 for the production of CD44-ICD. Subsequently, CD44-ICD translocates to the nucleus and induces the expression of genes associated with EMT and TAM polarization through the activation of transcriptional factors Smad2/3 and AP-1. Therefore, targeting TSG6 would provide a promising therapeutic strategy for LUAD treatment by modulating the tumor microenvironment.

## Supplementary Material

Supplementary figures and tables.
