# Supplementary material for: TSG6 promotes epithelial-mesenchymal transition and tumor-associated macrophage polarization through Smad2/3 and MAPK signaling by facilitating TSG6-CD44-TGFβR1 or EGFR complex formation
Source: Int J Biol Sci. 2025 Jul 24;21(11):4701–18. doi: 10.7150/ijbs.115097 (PMC12374808; doi:10.7150/ijbs.115097)
Supplement: Supplementary file 1 — Supplementary figures and tables. [file ijbsv21p4701s1.pdf]

Supplementary Information

TSG6 promotes epithelial-mesenchymal transition and tumor-associated macrophage polarization through Smad2/3 and MAPK signaling by facilitating TSG6-CD44-TGFβR1 or EGFR complex formation

Hyun-Ji Oh[§], Ga-Hong Min[§], Da-Eun Kim, Sol-bi Shin, and Hyungshin Yim[*]

Department of Pharmacy, College of Pharmacy, Institute of Pharmaceutical Science and Technology, Hanyang University, Ansan, Gyeonggi-do 15588, Republic of Korea

[*]Corresponding author

Hyungshin YIM

Address: Department of Pharmacy, College of Pharmacy, Institute of Pharmaceutical Science and Technology, Hanyang University, Ansan, Gyeonggi-do 15588, Republic of Korea

Phone: +82-31-400-5810

FAX: +82-31-400-5958

E-mail: hsyim@hanyang.ac.kr

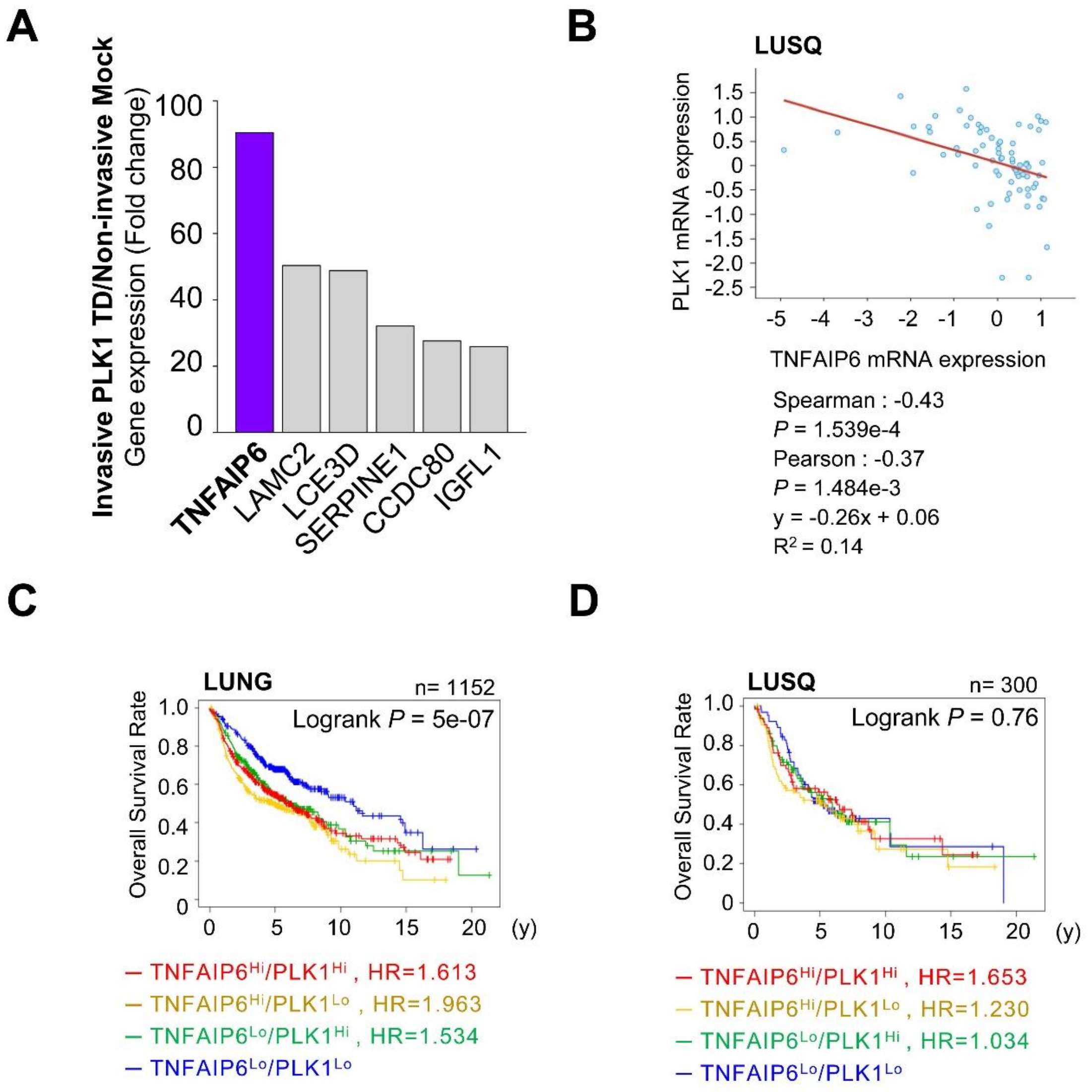

Figure S1. Correlation between *PLK1* and *TNFAIP6*. (A) Relative gene expression profile of top six genes in invasive cells expressing constitutively active T210D mutant of PLK1 (TD) cells, compared with non-invasive mock cells. **(B)** Analysis of spearman's and pearson's correlation coefficients between *TNFAIP6* and *PLK1* in lung squamous cell carcinoma (LUSQ) patients using cBioportal. **(C-D)** The overall survival times in lung cancer patients (n = 1152, Logrank P = 5e-07) **(C)** and LUSQ patients (n = 300, Logrank P = 0.76) **(D)** were analyzed according to *TNFAIP6* and *PLK1* expression levels using KM PLOTTER. High (Hi) and low (Lo) were generated by dividing patients according to their expression at the median cut-off.

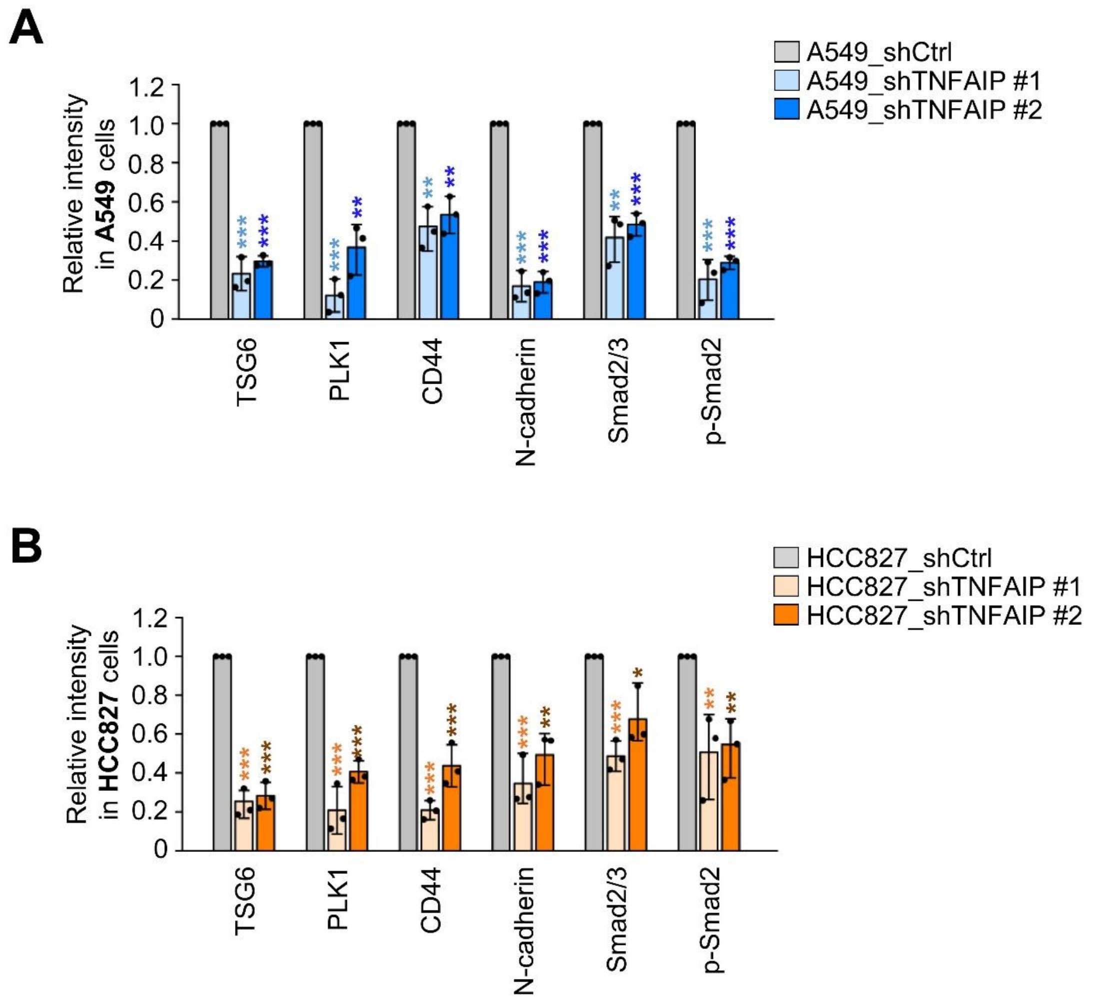

Figure S2. Depletion of *TNFAIP6* reduced the levels of mesenchymal factors. (A-B) The shRNA targeting at the position of 530-550 (shTNFAIP6#1) or 693-713 (shTNFAIP6#2) of human *TNFAIP6* was applied to A549 and HCC827 cells. The relative band intensities of **Fig. 4C** for TSG6, PLK1, CD44, N-cadherin, Smad2/3, and p-Smad2$^{S465/S467}$ were quantified in A549 **(A)** and HCC827 **(B)** cells using LI-COR Odyssey software.

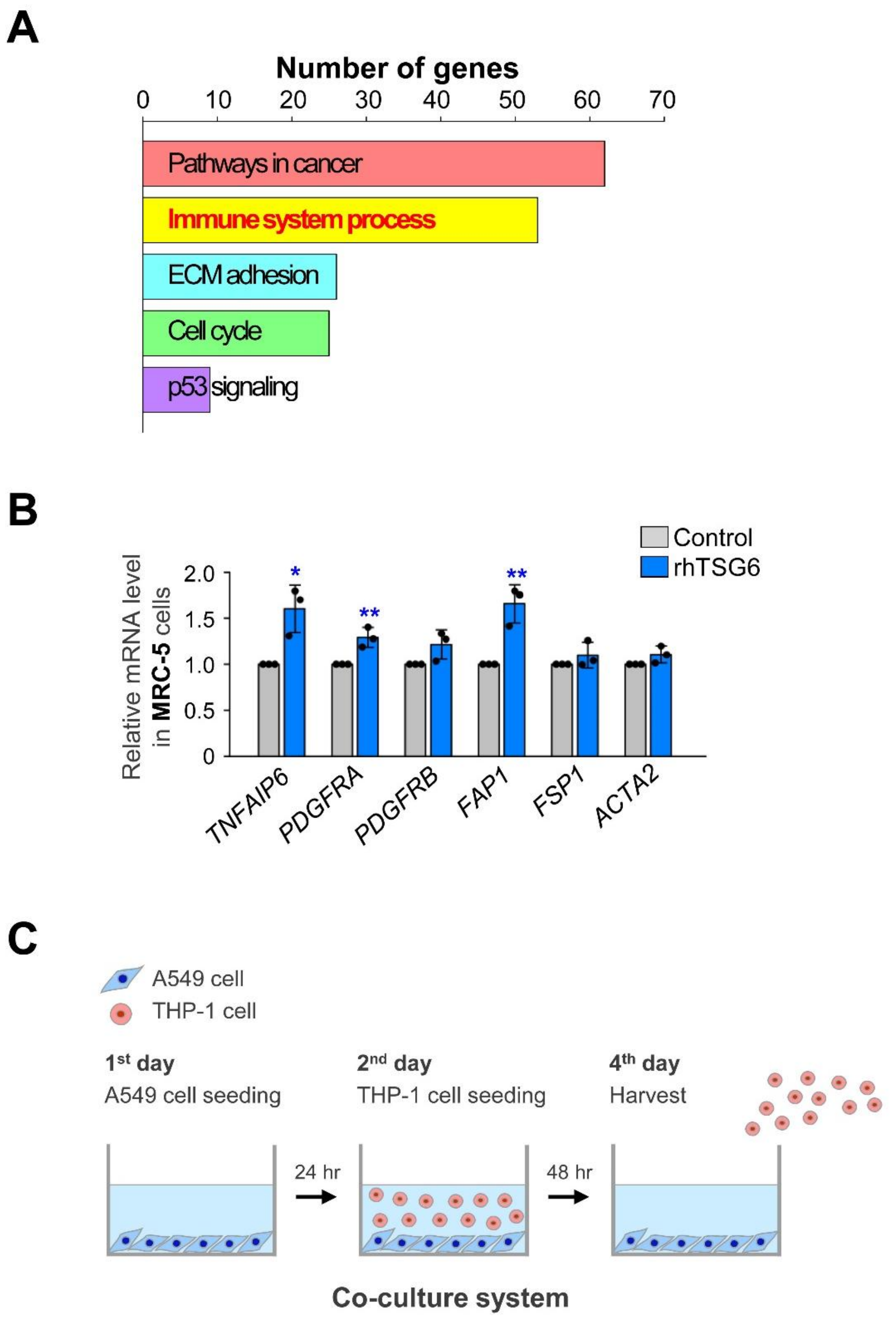

Figure S3. TSG6 did not much affect to the differentiation of cancer-associated fibroblast in the tumor microenvironments (A) Analysis of KEGG pathways regulated by active PLK1 extracted from the microarray data [12]. **(B)** MRC-5 lung fibroblast cells were treated with 200 ng/ml of rhTSG6 for 2 hours. qRT-PCR was performed for *TNFAIP6*, *PDGFRA*, *PDGFRB*,

FAP1, *FSP1*, and *ACTA2* expression in MRC-5 cells. *$p < 0.05$; ** $p < 0.01$ (n = 3). **(C)** The scheme of co-culture system between lung adenocarcinoma A549 cells and monocyte THP-1 cells.

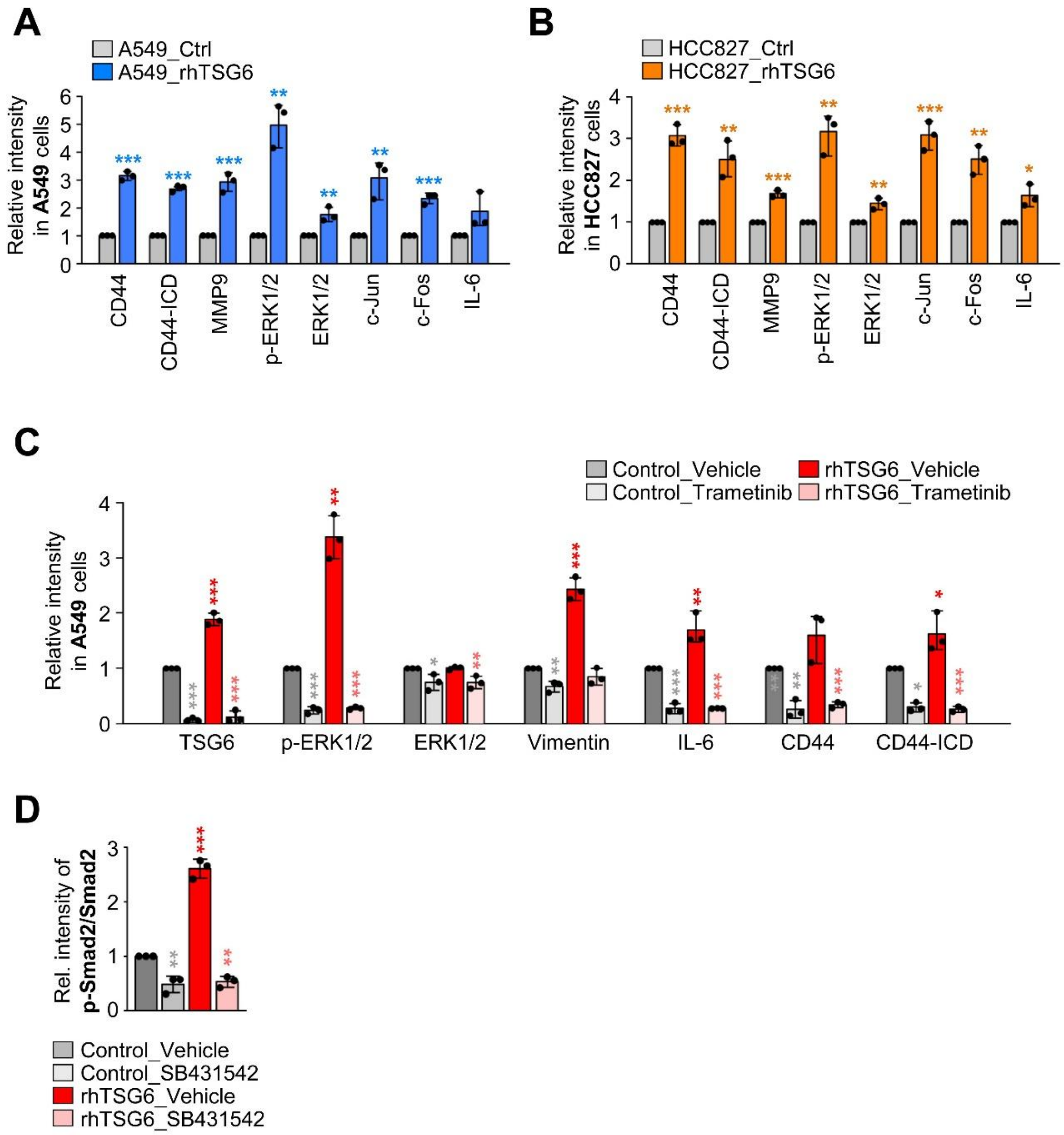

Figure S4. TSG6 mediated MAPK/ERK and TGFBR1/Smad signaling pathway for TAM polarization. **(A)** The shRNA targeting human *TNFAIP6* was applied to A549 and HCC827 cells. The relative band intensities of **Fig. 6A** for CD44, CD44-ICD, MMP9, p-ERK1/2, ERK1/2, c-Jun, c-Fos, and IL-6 were quantified in A549 **(A)** and HCC827 **(B)** cells using LI-COR Odyssey software. The relative band intensity was quantified using densitometry of Photoshop software. **(C)** A549 cells were treated with 10 μM trametinib (MEK1/2 inhibitor) for 48 hours and treated with 200 ng/ml of rhTSG6 for 2 hours. Immunoblot was performed to

measure the protein levels of TSG6, p-ERK1/2, ERK1/2, vimentin, IL-6, CD44, CD44-ICD, and GAPDH, and the relative band intensity values were analyzed in A549 cells. $*p < 0.05$; $** p < 0.01$; $*** p < 0.001$. (n = 3). **(D)** A549 cells were treated with 30 μM SB431542 (TGF-β receptor inhibitor) for 48 hours, and treated with 200 ng/ml of rhTSG6 for 2 hours. Immunoblot was performed to measure the protein levels of p-Smad2$^{S465/S467}$ and Smad2/3 in A549 cells. $**p < 0.01$; $***p < 0.001$ (n = 3). Data are presented as mean ± SD.

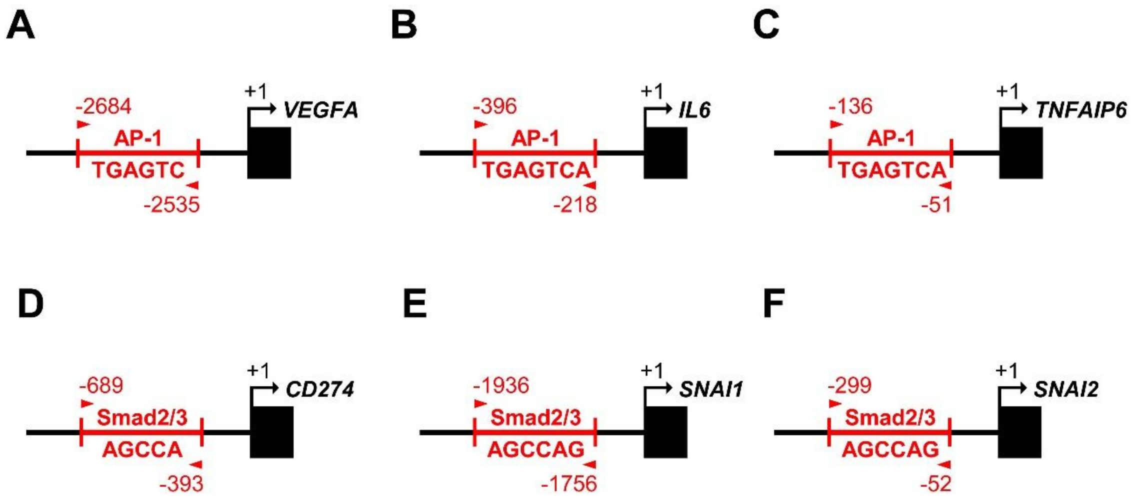

Figure S5. TSG6 facilitates the expression of genes involved in TAM polarization, EMT, and immune escape. The promoter regions of *VEGFA* **(A)**, *IL6* **(B)**, and *TNFAIP6* **(C)** for AP-1 (complex of c-Jun and c-Fos) binding and the promoter regions of *CD274* **(D)**, *SNAI1* **(E)**, and *SNAI2* **(F)** for Smad2/3 binding for ChIP assay in Figure 8D-I.

Supplementary Tables

Table S1. Cox regression analysis for survival of non-small cell lung cancer (NSCLC) and lung adenocarcinoma (LUAD) patients expressing TNFAIP6 and PLK1 of KM plot used in Figure 1.

Patients	Endpoint	Gene expression	Number of patients (n)	Hazard ratio (HR)	95% (CI)
LUNG	Overall Survival (OS) n=1152	TNFAIP6^{Hi}/PLK1^{Hi}	314	1.613	1.197~1.967
		TNFAIP6^{Hi}/PLK1^{Lo}	263	1.963	1.543~2.497
		TNFAIP6^{Lo}/PLK1^{Hi}	263	1.534	1.197~1.967
		TNFAIP6^{Lo}/PLK1^{Lo}	312	-	-
LUAD	Overall Survival (OS) n=656	TNFAIP6^{Hi}/PLK1^{Hi}	220	2.077	1.464~ 2.791
		TNFAIP6^{Hi}/PLK1^{Lo}	132	1.897	1.310~ 2.751
		TNFAIP6^{Lo}/PLK1^{Hi}	107	1.690	1.052~ 2.422
		TNFAIP6^{Lo}/PLK1^{Lo}	197	-	-
	Progression - Free Survival (PFS) n=115	TNFAIP6^{Hi}/PLK1^{Hi}	18	2.836	1.1917~6.748
		TNFAIP6^{Hi}/PLK1^{Lo}	18	2.494	0.9297~6.688
		TNFAIP6^{Lo}/PLK1^{Hi}	32	1.048	0.3974~2.762
		TNFAIP6^{Lo}/PLK1^{Lo}	47	-	-
LUSQ	Overall Survival (OS) n=300	TNFAIP6^{Hi}/PLK1^{Hi}	64	1.653	0.7964~1.899
		TNFAIP6^{Hi}/PLK1^{Lo}	86	1.230	0.6471~1.653
		TNFAIP6^{Lo}/PLK1^{Hi}	86	1.034	0.6574~1.588
		TNFAIP6^{Lo}/PLK1^{Lo}	64	-	-

Table S2. Sequences of forward (F) and reverse (R) primers used for qRT-PCR amplification.

Target Gene	Primer	Sequences
TNFAIP6	Forward	5'-GTGGCGTCTTTACAGATCC-3'
	Reverse	5'-CATCTCCACAGTATCTTCCC-3'
PLK1	Forward	5'-AAGAGATCCCGGAGGTCCTA-3'
	Reverse	5'-TCATTCAGGAAAAGGTTGCC-3'
CD44	Forward	5'-CAGTCTGACCAGCGTGAAAA-3'
	Reverse	5'-GGATTGATAGCCCTGTTGGA-3'
HAS2	Forward	5'-GTGTGTTCAGTGCATTAGTG-3'
	Reverse	5'-TAGGTGTTTCAGTAAGGCAC-3'
VIM	Forward	5'-GAGAACTTTGCCGTTGAAGC-3'
	Reverse	5'-GCTTCCTGTAGGTGGCAATC-3'
CDH1	Forward	5'-ACCACCTCCACAGCCACC-3'
	Reverse	5'-GTCCAGTTGGCACTCGCC-3'
CDH2	Forward	5'-ACAGTGGCCACCTACAAAGG-3'
	Reverse	5'-CCGAGATGGGGTTGATAATG-3'
VEGFA	Forward	5'-TTCCAGGAGTACCCTGATGA-3'
	Reverse	5'-TGAGGTTTGATCCGCATAAT-3'
IL6	Forward	5'-CAGACAGCCACTCACCTCTT-3'
	Reverse	5'-CTTTTTCAGCCATCTTTGGA-3'
IL4	Forward	5'-ACATTGTCACTGCAAATCGACACC-3'
	Reverse	5'-TGTCTGTTACGGTCAACTCGGTGC-3'
IL10	Forward	5'-AACCAAGACCCAGACATCAA-3'
	Reverse	5'-TGGCTTTGTAGATGCCTTTC-3'

TGFB1	Forward	5'-GGGACTATCCACCTGCAAGA-3'
	Reverse	5'-CCTCCTTGGCGTAGTAGTCG-3'
CD206	Forward	5'-ACTGCAAGCTTCACAATTCC-3'
	Reverse	5'-ATTTCAATTTGGGCTCATCA-3'
CD163	Forward	5'-TGATTCGGACTTCTCTCTGG-3'
	Reverse	5'-TGGCTACAAGTTCCTTCTGG-3'
IL12B	Forward	5'-GGAGCTGCTACACTCTCTGC-3'
	Reverse	5'-GATGAAGAAGCTGCTGGTGT-3'
iNOS	Forward	5'-TATCACAACCTCAGCAAGCA-3'
	Reverse	5'-AAAATCCCTTTGGCCTTATG-3'
GAPDH	Forward	5'-TAAAGGGCATCCTGGGCTACACT-3'
	Reverse	5'-TTACTCCTTGGAGGCCATGTAGG-3'
SMAD2	Forward	5'-GATCCTAACAGAACTTCCGCC-3'
	Reverse	5'-CACTTGTTTCTCCATCTTCACTG-3'

Table S3. Sequences of forward (F) and reverse (R) primers used for ChIP assay.

Target Gene	Primer	Sequences
VEGFA	Forward	5’-ATGGAAGGGAAGATGCCACA-3’
	Reverse	5’-TGTCACCGGCATTTACAACA-3’
IL6	Forward	5’-ACTTCGTGCATGACTTCAGC-3’
	Reverse	5’-GTGACGTCCTTTAGCATGGC-3’
TNFAIP6	Forward	5’-CTCCTTAGTTTTGGTTGCCA-3’
	Reverse	5’-GCAAGTACTCTCCAATGGCA-3’
CD274	Forward	5’-CTTAATCCTTAGGGTGGCAGA-3’
	Reverse	5’-AGGCGTCCCCCTTTCTGA-3’
SNAI1	Forward	5’-TCCTTCTGATGGGCGTGAAA-3’
	Reverse	5’-AACATCCCAGACCTTTCCCA-3’
SNAI2	Forward	5’-CTGCACCACATCTGGAAGCCAG-3’
	Reverse	5’-CCAATCACAGCTGAGAGGTTCAG-3’
